# Lean Mass Longitudinally Confounds Sedentary Time and Physical Activity With Blood Pressure Progression in 2513 Children

**DOI:** 10.1002/jcsm.13639

**Published:** 2024-11-13

**Authors:** Andrew O. Agbaje

**Affiliations:** ^1^ Institute of Public Health and Clinical Nutrition, School of Medicine, Faculty of Health Sciences University of Eastern Finland Kuopio Finland; ^2^ Children's Health and Exercise Research Centre, Department of Public Health and Sports Sciences, Faculty of Health and Life Sciences University of Exeter Exeter UK

**Keywords:** body composition, causality, hypertension, paediatrics, physical activity guidelines, prospective cohort study

## Abstract

**Background:**

Randomized controlled trials have reported no effect of moderate‐to‐vigorous physical activity (MVPA) on reducing blood pressure (BP) in youth, probably due to short trial durations. This study examined the longitudinal effect of sedentary time (ST), light PA (LPA) and MVPA on BP in 11‐year‐old children followed up for 13 years to determine the confounding and mediating role of body composition.

**Methods:**

Data included 2513 children from the Avon Longitudinal Study of Parents and Children (ALSPAC), UK birth cohort who had data on at least one time‐point measure of accelerometer‐based movement behaviour across the follow‐up and complete BP measures at ages 11, 15 and 24 years. Body composition was assessed with dual‐energy x‐ray absorptiometry at all time points. Multivariate adjusted generalized linear mixed‐effect model and structural equation causal mediation model analyses were conducted.

**Results:**

Among 2513 participants (61% female, mean [SD] age 11.72 [0.21] years), ST steadily increased from ~6 h/day in childhood (age 11 years) to ~9 h/day in young adulthood (age 24 years), whereas LPA and MVPA decreased, but BP had an inverted U‐shaped increase. In the longitudinal analysis, after full adjustment, a 1‐min cumulative ST from ages 11 to 24 years was positively associated with increased systolic BP (0.009 mmHg [95% CI 0.007–0.011]; *p* < 0.001) and diastolic BP. A 1‐min cumulative LPA was inversely associated with systolic BP (−0.007 mmHg [−0.009 to −0.004]; *p* < 0.001), but not diastolic BP. In isotemporal substitution analyses, longitudinal replacement of 10 min of ST with equal time in LPA during childhood, adolescence and young adulthood cumulatively decreased systolic BP by −2.63 mmHg [95% CI −3.17 to −2.08] (*p* < 0.0001) and diastolic BP by −1.93 mmHg [95% CI −2.36 to −1.50] (*p* < 0.0001). Replacing 10 min of ST with 10 min of MVPA had no statistically significant effect due to an absolute confounding effect of lean mass. The association of ST with systolic BP was fully mediated by increased lean mass (93% mediation). Increased total fat mass partially mediated (19%–27%) the inverse associations of cumulative MVPA with cumulative systolic and diastolic BP.

**Conclusions:**

Theoretically replacing 10 min/day spent sedentary with 10 min/day of LPA during growth from childhood through young adulthood may lower systolic BP by −3 mmHg and diastolic BP by −2 mmHg. Lean mass seems more significant than fat mass in the relations of ST and PA with BP and should be accounted for in future intervention studies in the paediatric and young adult population.

## 1 Introduction

Due to the lack of long‐term repeated body composition assessment with dual‐energy x‐ray absorptiometry, the distinct role of skeletal muscle mass and fat mass as either confounder or mediator remains unclear, and whether movement behaviours exert their effect on blood pressure (BP) directly or via metabolic or body composition pathways is not fully known [1, 2, 3, 4, 5, 6, 7, 8, 9, 10]. A few biological pathways on the associations between sedentary time (ST) and elevated BP identified in an experimental animal model and small sample‐sized human studies include altered lipid metabolism, insulin resistance, elevated inflammation and muscular atrophy [2, 3, 4, 5, 6, 7, 8, 9, 10].

Although the link between obesity and hypertension is well established in adults, several randomized controlled trials have failed to prove the efficacy of increasing physical activity (PA) and reducing obesity on lowering BP in paediatrics and young adult population [11, 12]. A meta‐analysis of 12 randomized controlled trials in 1266 children and adolescents conducted in 2003 concluded that short‐term exercise with a duration of 8–36 weeks did not reduce resting systolic and diastolic BP in children and adolescents [11]. Two decades later, another randomized clinical trial in youth did not find any effect of 60 min of aerobic training on BP after 16 and 52 weeks of follow‐up [12]. Hence, knowledge gaps exist in the paediatric population regarding whether exposure to long‐term light PA (LPA) and moderate‐to‐vigorous PA (MVPA) would lower BP and the quantitative estimate of the BP‐lowering effect of replacing time spent sedentary with time spent in LPA and MVPA during growth from childhood through young adulthood [1, 7, 9, 13].

The World Health Organization (WHO) estimates that half a billion new cases of physical inactivity‐related non‐communicable diseases would occur between 2020 and 2030 and 47% of those cases would result from hypertension [14]. Childhood and adolescent elevated BP and hypertension remain a global epidemic with a prevalence of 6%–12%, and higher childhood and adolescent BP has recently been associated with an increased risk of fatal and non‐fatal cardiovascular events in mid‐adulthood and premature cardiac damage in young adulthood [7, 13, 15, 16, 17, 18, 19, 20, 21, 22]. Importantly, the latest clinical practice guideline for screening and management of high BP in children and adolescents concluded that the recommendation of PA for lowering BP is weak and suggested a focus on lifestyle interventions such as weight reduction and nutrition [19].

Longitudinal evidence on the importance of timing for PA intervention in the paediatric population is limited and has implications for mounting effective childhood elevated BP prevention programs [1, 7, 13, 14, 16, 20]. The present study (1) examined the longitudinal associations of cumulative accelerometer‐measured ST, LPA and MVPA, with repeated measures of systolic and diastolic BP at ages 11, 15 and 24 years in the total cohort; (2) quantified the estimate of BP changes when an equal amount of time spent sedentary is replaced by time spent in LPA or MVPA also known as isotemporal substitution [23]; and (3) assessed the extent to which the longitudinal associations of movement behaviours with BP are mediated by total fat mass, lean mass, insulin resistance, lipid metabolism and inflammation using data from the Avon Longitudinal Study of Parents and Children (ALSPAC) birth cohort, England, UK.

## 2 Methods

### 2.1 Study Cohort

The unabridged method can be found in the Supporting Information. Data were from the ALSPAC birth cohort from Avon, England, UK, which investigates factors that influence childhood development and growth. In this study, 2513 participants with complete BP measures at 11‐, 15‐ and 24‐year clinic visits and at least one time‐point valid ST, LPA and MVPA measurements at either 11‐, 15‐ or 24‐year clinic visit were eligible for analyses (Figure S1). The excluded participants who had at least one time‐point measure of ST and PA and incomplete BP measures during the 13‐year‐long follow‐up study had similar characteristics with those included in the study (Table S1). Ethical approval for the study was obtained from the ALSPAC Ethics and Law Committee and the Local Research Ethics Committees. Informed consent for the use of data collected via questionnaires and clinics was obtained from participants following the recommendations of the ALSPAC Ethics and Law Committee at the time [24, 25, 26].

### 2.2 Exposures: ST and PA Assessment

ST, LPA and MVPA were assessed with ActiGraph (LLC, Fort Walton Beach, FL, USA) accelerometer worn on the waist for 7 consecutive days at 11‐ and 15‐year clinic visits, whereas at 24 years, movement behaviour was assessed using ActiGraph GT3X+ accelerometer device worn for 4 consecutive days [27]. Data are recorded as counts that result from summing postfiltered accelerometer values (raw data at 30 Hz) into 60‐s epoch units.

### 2.3 Outcomes: BP Measures

At 11‐ and 15‐year clinic visit, BP was measured using a Dinamap 9301 Vital Signs monitor. The child was first given a simple explanation of what would happen in the session using the analogy of an inflating balloon to explain the action of the cuff. Two cuffs were used depending on the size of the child's upper arm circumference (ideally the right arm was used): If < 23 cm, a small adult‐size cuff was used, and if ≥ 23 cm, an adult cuff was used. Two readings of systolic and diastolic BP were recorded, and the mean of each was calculated for analysis. If the child's BP was 140/90 mmHg or more the parents were given a letter to take to their physician [28]. At 24‐year clinic visit, BP readings were taken using an Omron M6 upper arm BP.

### 2.4 Confounders and Covariates: Anthropometry, Body Composition, Cardiometabolic, Socio‐economic and Lifestyle Factors

Anthropometry (height and weight) of participants at ages 11, 15, and 24 years were assessed in line with standard protocols, and body mass index was computed as weight in kilograms per height in metres squared [28, 29]. Body composition (total body fat mass and total body lean mass) was assessed using dual‐energy x‐ray absorptiometry scanner at 11‐, 15‐ and 24‐year clinic visits as previously described [28, 29, 30]. Heart rate was measured with semi‐automated digital monitors at ages 11, 15, and 24 years as previously detailed [28, 29]. Using standard protocols, fasting blood samples at ages 15, 17 and 24 years were collected, spun and frozen at −80°C, and a detailed assessment of fasting glucose, insulin, high‐sensitivity C‐reactive protein (hsCRP), low‐density lipoprotein cholesterol (LDL‐c), high‐density lipoprotein cholesterol (HDL‐c) and triglycerides. The homeostatic model assessment of insulin resistance (HOMA‐IR) was computed from (fasting insulin× fasting glucose/22.5). At the 17‐year clinic visit, participants were briefly asked about their personal and family (mother, father and siblings) medical history such as a history of hypertension, diabetes, high cholesterol and vascular disease.

The participant's mother's socio‐economic status was grouped according to the 1991 British Office of Population and Census Statistics classification. Questionnaires to assess smoking behaviour were administered at the 13‐, 15‐ and 24‐year clinic visits. A specific question regarding whether participants smoked in the last 30 days was used as an indicator of current smoking status.

### 2.5 Statistical Analysis

Cohort descriptive characteristics were summarized as means and standard deviation, medians and interquartile ranges or frequencies and percentages.

#### 2.5.1 Analyses of Longitudinal Associations (Single and Partition Models)

We examined the separate longitudinal associations of each of the 13‐year ST, LPA and MVPA progression (11–24 years) with each of systolic and diastolic BP measured at ages 11, 15 and 24 years using generalized linear mixed‐effect models (GLMM). Although the GLMM is robust for handling missing at random predictor and covariate data, we elected to additionally conduct 20 cycles of multiple imputations to account for missing data. The GLMM accounted for baseline ST, LPA, MVPA predictors, BP outcomes and covariates and their repeated measures. For ST, LPA and MVPA continuous variable analyses, Model 1 was unadjusted. Model 2 was adjusted for sex and other time‐varying covariates measured at both baseline and follow‐up such as age, LDL‐c, triglyceride, hsCRP, HDL‐c, heart rate, glucose, insulin, smoking status, family history of hypertension/diabetes/high cholesterol/vascular disease, socio‐economic status, fat mass and lean mass. Model 3 was an additional adjustment for either ST or LPA depending on the predictor. Model 4 was an additional adjustment for either LPA or MVPA depending on the predictor. Model 5 was the exclusion of lean mass from Model 4 due to its strong relationship with blood pressure [28]. For categorical MVPA variable analyses, all the above‐listed covariates were adjusted for in Model 1, whereas in Model 2 lean mass was excluded. A different GLMM was conducted for each sex, and sex‐specific analyses were not adjusted for sex.

#### 2.5.2 Isotemporal Substitution Longitudinal Analyses

To simulate the theoretical effect of replacing time spent sedentary with an equal amount of time spent in LPA and MVPA, all activity variables (LPA and MVPA), except for ST, were entered into the GLMM simultaneously, along with a total wear time variable and the above‐listed covariates. More details can be found in the Supporting Information.

#### 2.5.3 Mediation Path Longitudinal Analyses

Lastly, mediating path analyses using structural equation models separately examined the mediating role of cumulative fat mass, lean mass, insulin resistance, lipids and hsCRP on the longitudinal associations of cumulative ST, LPA or MVPA with each of systolic and diastolic BP.

Collinearity diagnoses were performed and accepted results with a variance inflation factor < 2, considered differences and associations with a two‐sided *p*‐value < 0.05 as statistically significant, and conclusions were made based on effect estimates and their confidence intervals (CI). Covariates were identified based on previous studies and are portrayed in the directed acyclic graph (Figure S2). We applied Sidak correction for potential multiple comparisons. Analyses involving 20% of a sample of 10 000 ALSPAC children at 0.8 statistical power, 0.05 alpha and two‐sided *p*‐value would show a minimum detectable effect size of 0.062 standard deviations if they had relevant exposure for a normally distributed quantitative variable. All statistical analyses were performed using SPSS Statistics software, Version 27.0 (IBM Corp, Armonk, NY, USA), and mediation analysis structural equation modelling was conducted using IBM AMOS Version 27.0.

## 3 Results

**FIGURE 1:**
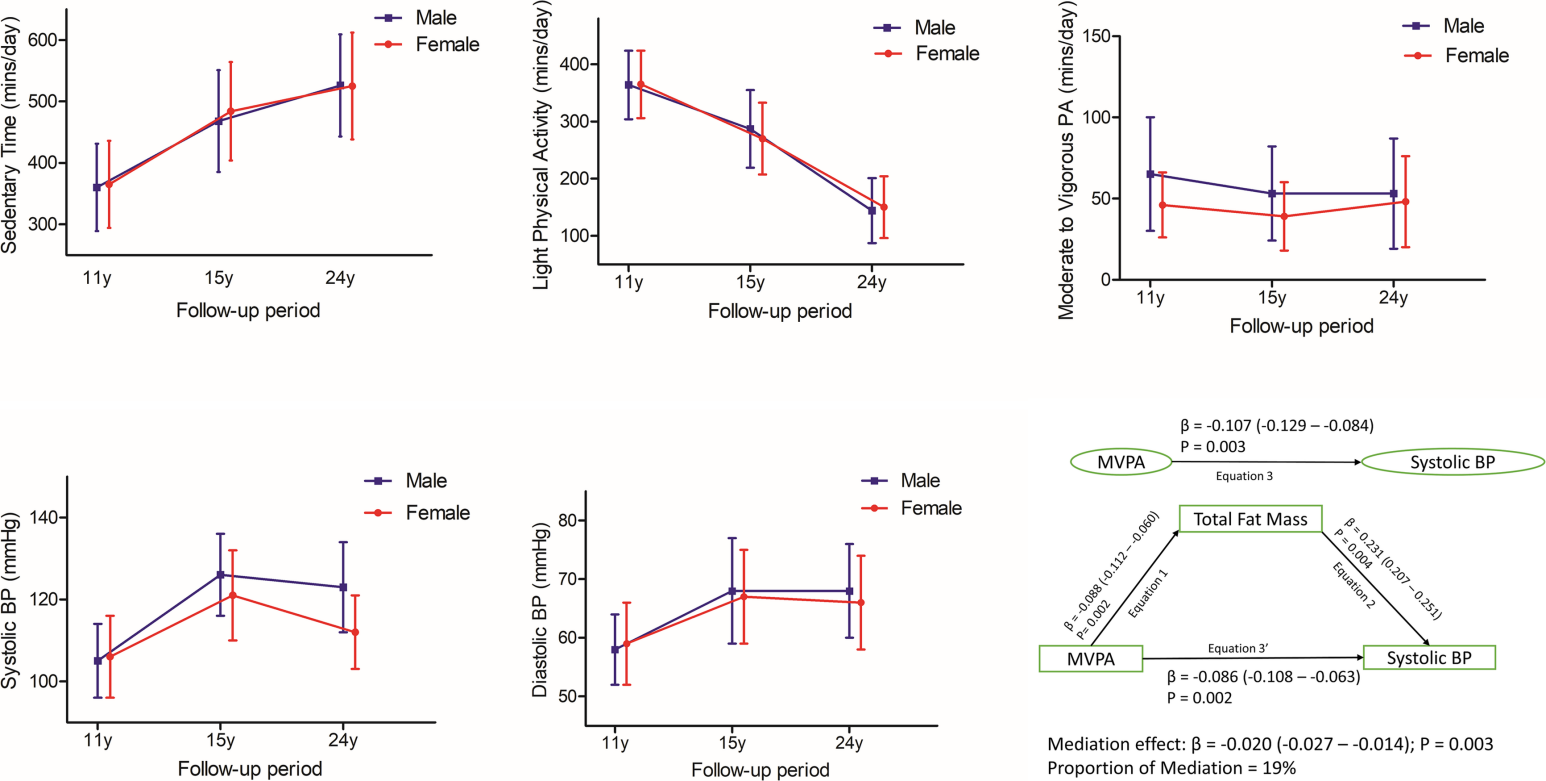
Trajectories of movement behaviours and blood pressure (mean ± SD) from ages 11 through 24 years and longitudinal associations between MVPA and systolic blood pressure with the mediating effect of total fat mass in 2513 children. When the magnitude of the longitudinal association between the predictor and outcome is decreased upon inclusion of a third variable, a mediation is confirmed, BP, blood pressure; MVPA, moderate‐to‐vigorous physical activity. Mediation structural equation model estimating natural direct and indirect effects was adjusted for sex, family history of hypertension/diabetes/high cholesterol/vascular disease and socio‐economic status, in addition to time‐varying covariates such as age, high‐sensitivity C‐reactive protein, heart rate, smoking status, sedentary time, light physical activity, high‐density lipoprotein cholesterol, low‐density lipoprotein cholesterol, triglyceride, lean mass, glucose and insulin. *β* is standardized regression coefficient. Two‐sided *p*‐value < 0.05 was considered statistically significant.

From ages 11 through 24 years, BP increased in an inverted U‐shape in both males and females (Table 1 and Figure 1). ST increased from 6 h/day in childhood to 9 h/day in young adulthood, whereas LPA decreased from ages 11 through 24 years in both males and females (Table 1 and Figure 1). MVPA in min/day decreased in both males and females, with males accruing more ≥ 60 min/day of MVPA across the 13‐year follow‐up period than females (Table 1 and Figure 1). Altogether, 2513 participants who had complete systolic and diastolic BP measures at ages 11, 15, and 24 years with at least one time‐point measure of ST, LPA and MVPA were included (Figure S1). The excluded participants who had at least one time‐point measure of movement behaviour but incomplete three time‐point BP measures during the 13‐year‐long follow‐up study had similar baseline characteristics with those included in the study (Table S1). Other characteristics are described in Table 1.

**TABLE 1:** Descriptive characteristics of 2513 participants who had at least one time‐point measure of movement behaviour at either 11, 15 or 24 years and complete blood pressure measures at 11‐, 15‐ and 24‐year clinic visits.

Age at clinic visits/follow‐up	11 years			15 years			24 years		
Variables	Male (*n* = 976)	Female (*n* = 1537)	*p*	Male (*n* = 976)	Female (*n* = 1537)	*p*	Male (*n* = 976)	Female (*n* = 1537)	*p*
Anthropometry									
Age at clinic visit (years), mean (SD)	11.71 (0.20)	11.72 (0.21)	0.297	15.38 (0.22)	15.41 (0.26)	0.004	24.52 (0.77)	24.43 (0.75)	0.005
Height (m), mean (SD)	1.51 (0.07)	1.51 (0.07)	0.097	1.75 (0.07)	1.65 (0.06)	< 0.0001	1.80 (0.07)	1.66 (0.06)	< 0.0001
Weight^a^ (kg)	41.20 (12.2)	42.40 (12.6)	0.001	62.10 (13.4)	56.90 (12.1)	< 0.0001	78.25 (18.58)	64.65 (17.17)	< 0.0001
Body composition									
Total fat mass^a^ (kg)	8.39 (8.0)	11.07 (8.10)	< 0.0001	8.45 (7.87)	17.05 (9.21)	< 0.0001	18.25 (11.71)	22.06 (12.21)	< 0.0001
Lean mass^a^ (kg)	29.92 (5.40)	28.79 (6.35)	< 0.0001	49.70 (8.13)	36.73 (5.18)	< 0.0001	56.25 (10.29)	40.72 (6.70)	< 0.0001
Body mass index^a^ (kg/m^2^)	17.99 (3.99)	18.53 (4.10)	0.001	20.25 (3.68)	21.05 (3.95)	< 0.0001	24.13 (4.99)	23.47 (5.78)	0.094
Vascular measures									
Heart rate (beat/min), mean (SD)	74 (10)	78 (11)	< 0.0001	72 (12)	77 (12)	< 0.0001	65 (11)	68 (10)	< 0.0001
Systolic blood pressure (mmHg), mean (SD)	105 (9)	106 (10)	0.003	126 (10)	121 (11)	< 0.0001	123 (11)	112 (9)	< 0.0001
Diastolic blood pressure (mmHg), mean (SD)	58 (6)	59 (7)	0.023	68 (9)	67 (8)	< 0.0001	68 (8)	66 (8)	< 0.0001
Lifestyle and sociodemographic factors									
Smoked in the last 30 days (*n*, %)	< 7 (0.4)	35 (2.4)	< 0.0001	87 (9.2)	246 (16.2)	< 0.0001	267 (27.8)	393 (25.8)	0.284
Family history of H‐D‐C‐V (*n*, %)	246 (29.2)	409 (26.6)	0.632	NA			NA		
Sedentary time (min/day), mean (SD)	360 (71)	365 (71)	0.092	468 (83)	484 (80)	< 0.0001	526 (83)	525 (87)	0.939
Light physical activity (min/day), mean (SD)	364 (60)	365 (59)	0.722	287 (68)	270 (63)	< 0.0001	144 (57)	150 (54)	0.209
MVPA (min/day), mean (SD)	65 (35)	46 (20)	< 0.0001	53 (29)	39 (21)	< 0.0001	53 (34)	48 (28)	0.032
MVPA < 40 min/day (*n*, %)	173 (18.7)	315 (21.8)	< 0.0001	185 (34.7)	501 (56.4)	< 0.0001	90 (40.5)	189 (46.0)	0.179
MVPA 40–<60 min/day (*n*, %)	263 (28.4)	513 (35.4)	< 0.0001	163 (30.6)	242 (27.2)	< 0.0001	58 (26.1)	102 (24.8)	0.179
MVPA ≥ 60 min/day (*n*, %)	491 (53.0)	620 (42.8)	< 0.0001	185 (34.7)	146 (16.4)	< 0.0001	74 (33.3)	120 (29.2)	0.179
Ethnicity: White (*n*, %)	877 (96.3)	1367 (96.3)	0.999	NA			NA		
Maternal socio‐economic status (*n*, %)			0.189	NA			NA		
Professional	49 (10.2)	39 (5.5)							
Managerial and technical	192 (40.1)	287 (40.4)							
Skilled non‐manual	155 (32.4)	259 (36.4)							
Skilled manual	< 7 (1.3)	15 (2.1)							
Partly skilled	64 (13.4)	90 (12.7)							
Unskilled	13 (2.7)	21 (3.0)							
Fasting plasma metabolic indices	15 years			17 years			24 years		
High‐density lipoprotein (mmol/L), mean (SD)	1.22 (0.26)	1.36 (0.29)	< 0.0001	1.19 (0.26)	1.36 (0.32)	< 0.0001	1.40 (0.37)	1.65 (0.42)	< 0.0001
Low‐density lipoprotein (mmol/L), mean (SD)	1.99 (0.52)	2.16 (0.56)	< 0.0001	2.01 (0.57)	2.22 (0.63)	< 0.0001	2.48 (0.78)	2.43 (0.75)	0.154
Triglyceride^a^ (mmol/L)	0.72 (0.36)	0.77 (0.39)	< 0.0001	0.74 (0.33)	0.75 (0.37)	0.564	0.89 (0.59)	0.79 (0.42)	< 0.0001
Glucose (mmol/L), mean (SD)	5.30 (0.39)	5.14 (0.39)	< 0.0001	5.14 (0.39)	4.91 (0.38)	< 0.0001	5.46 (0.76)	5.20 (0.51)	< 0.0001
Insulin^a^ (mU/L)	8.17 (4.87)	9.70 (5.43)	< 0.0001	5.87 (3.76)	7.40 (4.28)	< 0.0001	7.02 (5.11)	7.87 (5.58)	< 0.0001
High‐sensitivity C‐reactive protein^a^ (mg/L)	0.37^a^ (0.64)	0.37 (0.65)	0.246	0.43 (0.64)	0.63 (1.33)	< 0.0001	0.64 (1.13)	1.02 (2.11)	< 0.0001

*Note:* The values are means (standard deviations) and median (interquartile range) except for lifestyle factors and ethnicity. Differences between sexes were tested using Student's *t*‐test for normally distributed continuous variables, Mann–Whitney *U* test for skewed continuous variables, chi‐square test for dichotomous variables and analysis of covariance for the multicategory variable. A two‐sided *p*‐value < 0.05 is considered statistically significant and is bolded.

Abbreviations: H‐D‐C‐V, hypertension/diabetes/high cholesterol/vascular disease; MVPA, moderate‐to‐vigorous physical activity; NA, not available/applicable.

^a^ Median (interquartile range).

### 3.1 Longitudinal Associations of ST, LPA and MVPA With BP *(Single and Partition Models)*

In summary, ST from childhood increased systolic and diastolic BP, whereas LPA decreased systolic BP and MVPA had no effect on BP as detailed below. In the total cohort, males and females, cumulative ST was directly associated with increased systolic and diastolic BP from ages 11 to 24 years after full adjustments for cardiometabolic and lifestyle factors including LPA and MVPA (Tables 2 and S2). Cumulative LPA was inversely associated with increased systolic BP but not diastolic BP progression after full adjustments in the total cohort and in females (Tables 2 and S2). Cumulative MVPA was inversely associated with increased systolic BP only after full adjustments for covariates that excluded cumulative lean mass (Table 2). Additional adjustment for lean mass significantly attenuated the relationship between MVPA and systolic BP. Cumulative MVPA was directly associated with increased diastolic BP after full adjustment in the total cohort and in males (Tables 2 and S2). Persistent ≥ 60 min/day of MVPA was associated with increased diastolic BP but not systolic BP after full adjustment (Table 2). After excluding lean mass, persistent ≥ 60 min/day of MVPA was inversely associated with cumulative systolic BP but not diastolic BP.

**TABLE 2:** Longitudinal associations of cumulative sedentary time and physical activity with blood pressure progression from ages 11 through 24 years among participants with at least one time‐point movement behaviour measure and complete three time‐point blood pressure measures.

*N* = 2513	Systolic blood pressure		Diastolic blood pressure	
	*β (95% CI)*	*p*	*β (95% CI)*	*p*
Continuous cumulative predictor variables from ages 11 to 24 years				
Sedentary time (min/day)				
Model 1	0.043 (0.041–0.045)	**< 0.0001**	0.030 (0.029–0.031)	**< 0.0001**
Model 2	0.011 (0.009–0.013)	**< 0.0001**	0.009 (0.007–0.011)	**< 0.0001**
Model 3	0.009 (0.007–0.011)	**< 0.001**	0.009 (0.007–0.010)	**< 0.0001**
Model 4	0.009 (0.007–0.011)	**< 0.001**	0.009 (0.007–0.011)	**< 0.0001**
Model 5	0.032 (0.029–0.035)	**< 0.0001**	0.018 (0.016–0.020)	**< 0.0001**
Light physical activity (min/day)				
Model 1	−0.031 (−0.033 to −0.029)	**< 0.0001**	−0.026 (−0.027 to −0.024)	**< 0.0001**
Model 2	−0.012 (−0.015 to −0.010)	**< 0.0001**	−0.006 (−0.008 to −0.004)	**< 0.001**
Model 3	−0.007 (−0.009 to −0.004)	**< 0.001**	−0.001 (−0.003–0.001)	0.332
Model 4	−0.007 (−0.009 to −0.004)	**< 0.001**	−0.001 (−0.003–0.001)	0.284
Model 5	−0.004 (−0.007–0.000)	0.059	−0.0001 (−0.002–0.002)	0.968
Moderate‐to‐vigorous physical activity (min/day)				
Model 1	−0.067 (−0.078 to −0.056)	**< 0.0001**	−0.024 (−0.031 to −0.017)	**< 0.001**
Model 2	−0.008 (−0.015 to −0.001)	**0.033**	0.000 (−0.005–0.006)	0.859
Model 3	0.000 (−0.008–0.008)	0.999	0.007 (0.002–0.013)	**0.009**
Model 4	0.000 (−0.007–0.008)	0.919	0.007 (0.002–0.013)	**0.008**
Model 5	−0.023 (−0.033 to −0.013)	**< 0.001**	−0.001 (−0.007–0.004)	0.623
Categorical cumulative predictor variable from ages 11 to 24 years				
Moderate‐to‐vigorous physical activity				
Model 1	< 40 min/day of MVPA as reference			
40–<60 min/day	0.094 (−0.435–0.248)	0.592	0.032 (−0.245–0.309)	0.820
≥ 60 min/day	0.055 (−0.382–0.492)	0.805	0.355 (0.006–0.705)	**0.046**
Model 2	< 40 min/day of MVPA as reference			
40–<60 min/day	−0.221 (−0.652–0.209)	0.314	−0.015 (−0.308–0.277)	0.918
≥ 60 min/day	−1.435 (−2.004 to −0.867)	**< 0.001**	−0.204 (−0.576–0.168)	0.283

*Note:* For continuous variable analyses, Model 1 was unadjusted. Model 2 was adjusted for sex and other time‐varying covariates measured at both baseline and follow‐up such as age, low‐density lipoprotein cholesterol, triglyceride, high‐sensitivity C‐reactive protein, high‐density lipoprotein cholesterol, heart rate, glucose, insulin, smoking status, family history of hypertension/diabetes/high cholesterol/vascular disease, socio‐economic status, fat mass and lean mass. Model 3 was an additional adjustment for either sedentary time or light physical activity depending on the predictor. Model 4 was an additional adjustment for either light physical activity or moderate‐to‐vigorous physical activity depending on the predictor. Model 5 was exclusion of lean mass from Model 4 due to its strong relationship with blood pressure. For categorical predictor variable analyses, all the above‐listed covariates were adjusted for in Model 1, whereas in Model 2, lean mass was excluded. Skewed covariates were logarithmically transformed. Unstandardized regression coefficients *(β)* were computed from the generalized linear mixed‐effect model for repeated measures.  *P*‐values < 0.05 are considered statistically significant and are bolded. Multiple testing was corrected with Sidak correction. Multiple imputations were used to account for missing variables. For continuous variable predictors (ST, LPA and MVPA), a 1‐min change in movement behaviour is associated with a 1‐mmHg change in blood pressure. For categorical variable predictor (MVPA), time spent in a category in relation to the reference is associated with 1‐mmHg change in blood pressure.

Abbreviation: CI, confidence interval.

For sensitivity analyses, findings among 1556 participants who had at least two time‐point measures of movement behaviour and complete BP measures were consistent with the main results from 2513 participants who had at least one time‐point movement behaviour and complete BP measures (Table S3). In the complete case analysis conducted among 427 participants who had all predictor, outcome and covariates at all time points, cumulative ST was longitudinally associated with systolic and diastolic BP from ages 11 to 24 years in the fully adjusted model consistent with the analyses in 2513 and 1556 participants (Table S4). However, cumulative LPA or MVPA was not associated with systolic and diastolic BP after full adjustments (Table S4).

### 3.2 Longitudinal Associations of ST, LPA and MVPA With BP *(Compositional Data Analysis)*

Using compositional data analyses, cumulative ST relative to cumulative LPA and MVPA was longitudinally associated with increased systolic BP but not diastolic BP only when lean mass was excluded from the fully adjusted model (Table S5). Cumulative LPA relative to cumulative ST and MVPA was longitudinally associated with decreased systolic and diastolic BP in the fully adjusted model with or without lean mass exclusion (Table S5). Cumulative MVPA relative to cumulative ST and LPA was not associated with either systolic or diastolic BP after full adjustment. However, cumulative MVPA relative to ST was associated with decreased systolic BP in the fully adjusted model only when lean mass was excluded (Table S5).

### 3.3 Isotemporal Longitudinal Substitution of 10 or 30 min of ST, With an Equal Amount of LPA and MVPA in the Associations With BP

A 10‐min replacement of ST at each time‐point 11‐, 15‐ and 24‐year follow‐up with 10 min of LPA was associated with a −2.6 mmHg (−3.2 to −2.1) cumulative decrease in systolic BP and −1.9 mmHg (−2.4 to −1.5) cumulative decrease in diastolic BP over 13‐year follow‐up after full adjustments for cardiometabolic and lifestyle factors (Table 3). A 10‐min replacement of ST at each time‐point 11‐, 15‐ and 24‐year follow‐up with 10 min of MVPA was not associated with a decrease in either systolic or diastolic BP after full adjustments (Table 3).

**TABLE 3:** Isotemporal longitudinal substitution of 10 min of sedentary time with equal time spent in physical activity in relation to blood pressure progression from ages 11 through 24 years among participants with at least one‐time‐point movement behaviour measure and complete three time‐point blood pressure measures.

	10‐min reallocation at ages 11, 15 and 24 years				30‐min reallocation at ages 11, 15 and 24 years			
	Systolic blood pressure		Diastolic blood pressure		Systolic blood pressure		Diastolic blood pressure	
*N* = 2513	*β (95% CI)*	*p*	*β (95% CI)*	*p*	*β (95% CI)*	*p*	*β (95% CI)*	*p*
Model 1								
Sedentary time	Dropped		Dropped		Dropped		Dropped	
LPA	−2.627 (−3.172 to −2.081)	**< 0.0001**	−1.927 (−2.355 to −1.499)	**< 0.0001**	−0.876 (−1.057 to −0.694)	**< 0.0001**	−0.642 (−0.785 to −0.500)	**< 0.0001**
MVPA	−1.653 (−3.425–0.119)	0.068	0.234 (−1.019–1.486)	0.715	−0.551 (−1.142–0.040)	0.068	0.078 (−0.340–0.495)	0.715
Model 2								
Sedentary time	Dropped		Dropped		Dropped		Dropped	
LPA	−7.632 (−8.281 to −6.982)	**< 0.0001**	−3.796 (−4.234 to −3.359)	**< 0.0001**	−2.544 (−2.760 to −2.327)	**< 0.0001**	−1.265 (−1.411 to −1.120)	**< 0.0001**
MVPA	−11.331 (−13.526 to −9.136)	**< 0.0001**	−3.379 (−4.716 to −2.042)	**< 0.001**	−3.777 (−4.509 to −3.045)	**< 0.0001**	−1.126 (−1.572 to −0.681)	**< 0.001**

*Note:* Model 1 was isotemporal substitution of 10 or 30 min of sedentary time at ages 11, 15 and 24 years with an equal amount of time spent in light physical activity and moderate to vigorous physical activity and further adjusted for sex and other time‐varying covariates measured at both baseline and follow‐up such as age, low‐density lipoprotein cholesterol, triglyceride, high‐sensitivity C‐reactive protein, high‐density lipoprotein cholesterol, heart rate, glucose, insulin, smoking status, family history of hypertension/diabetes/high cholesterol/vascular disease, socio‐economic status, fat mass, lean mass and total activity wear time. Model 2 was Model 1 without adjusting for lean mass. Skewed covariates were logarithmically transformed. Unstandardized regression coefficients *(β)* were computed from the generalized linear mixed‐effect model for repeated measures. *P*‐values < 0.05 are considered statistically significant and are bolded. Multiple testing was corrected with Sidak correction. Multiple imputations were used to account for missing variables. A 10‐ or 30‐min replacement of movement behaviour is associated with a unit in mmHg change in blood pressure.

Abbreviation: CI, confidence interval.

However, after excluding lean mass, a 10‐min replacement of ST with 10 min of LPA was associated with a −7.6 mmHg cumulative decrease in systolic BP and a −3.8 mmHg cumulative decrease in diastolic BP. Likewise, after excluding lean mass, a 10‐min replacement of ST with 10 min of MVPA was associated with a −11.3 mmHg cumulative decrease in systolic BP and a −3.4 mmHg cumulative decrease in diastolic BP (Table 3). The isotemporal substitution results were consistent when 30 min of ST was replaced with either an equal time spent in LPA or MVPA in the fully adjusted models or after lean mass exclusion. However, the reduction in BP after replacing ST with 10 min of LPA was threefold greater than the reduction in BP after 30 min of isotemporal replacement (−2.7 vs. −0.9 mmHg) (Table 3).

### 3.4 Mediating or Suppressing Effects of Total Fat Mass, Lean Mass, Insulin Resistance, Lipids and Inflammation in the Longitudinal Associations of ST, LPA and MVPA With Systolic and Diastolic BP

Body composition explains the effect of movement behaviour on BP as detailed below. Cumulative HDL‐c, triglyceride, hsCRP and total fat mass had no statistically significant mediating effect on the positive associations of cumulative ST with cumulative systolic and diastolic BP (Table 4). Cumulative insulin resistance partially suppressed (3.5%–6.5%) the positive associations of increased ST with cumulative systolic and diastolic BP (Table 4). Cumulative lean mass almost fully mediated (92.7%) the positive associations of increased ST with cumulative systolic BP and partly mediated (26.5%) the positive associations of ST with diastolic BP (Table 4).

**TABLE 4:** Mediating or suppressing role of cumulative fat mass, lean mass and fasting insulin resistance, lipids and inflammation on the longitudinal associations of cumulative sedentary behaviour and blood pressure progression from ages 11 through 24 years of 2513 participants.

Cumulative sedentary time	Cumulative systolic blood pressure from ages 11 to 24 years						
	Total effect		Direct effect		Indirect effect		Mediation or suppression (%)
Mediators	*β (95% CI)*	*p*	*β (95% CI)*	*p*	*β (95% CI)*	*p*	
HDL	0.368 (0.349–0.388)	0.002	0.365 (0.347–0.384)	0.002	0.003 (−0.003–0.009)	0.297	0.8
LDL	0.319 (0.298–0.338)	0.003	0.320 (0.300–0.339)	0.003	−0.001 (−0.003–0.000)	**0.021**	**0.3 suppression**
Triglyceride	0.312 (0.292–0.330)	0.003	0.313 (0.293–0.332)	0.003	−0.001 (−0.005–0.002)	0.395	0.3
Insulin resistance	0.324 (0.304–0.342)	0.003	0.327 (0.308–0.346)	0.003	−0.004 (−0.007–0.000)	**0.021**	**6.5 suppression**
High‐sensitivity CRP	0.311 (0.291–0.333)	0.002	0.311 (0.292–0.332)	0.002	0.000 (−0.002–0.003)	0.970	0
Fat mass	0.270 (0.245–0.293)	0.002	0.266 (0.241–0.289)	0.002	0.004 (0.000–0.008)	0.050	1.5
Lean mass	0.123 (0.099–0.150)	0.002	0.009 (−0.013–0.035)	0.461	0.114 (0.100–0.129)	**0.002**	**92.7 mediation**

Cumulative sedentary time	Cumulative diastolic blood pressure from ages 11 to 24 years						
	Total effect		Direct effect		Indirect effect		Mediation or suppression (%)
Mediators	*β (95% CI)*	*p*	*β (95% CI)*	*p*	*β (95% CI)*	*p*	
HDL	0.369 (0.350–0.388)	0.003	0.368 (0.350–0.387)	0.002	0.001 (−0.001–0.004)	0.297	0.3
LDL	0.334 (0.315–0.353)	0.002	0.336 (0.318–0.356)	0.002	−0.003 (−0.005–0.000)	**0.015**	**0.9 suppression**
Triglyceride	0.334 (0.316–0.354)	0.002	0.335 (0.317–0.354)	0.003	−0.001 (−0.005–0.002)	0.409	0.3
Insulin resistance	0.343 (0.326–0.363)	0.002	0.355 (0.337–0.374)	0.003	−0.012 (−0.016 to −0.008)	**0.003**	**3.5 suppression**
High‐sensitivity CRP	0.327 (0.310–0.348)	0.002	0.327 (0.309–0.347)	0.002	0.000 (−0.004–0.004)	0.970	0
Fat mass	0.280 (0.261–0.303)	0.001	0.275 (0.256–0.298)	0.001	0.005 (0.000–0.010)	**0.047**	**1.8 mediation**
Lean mass	0.238 (0.213–0.259)	0.003	0.175 (0.148–0.198)	0.003	0.063 (0.054–0.072)	**0.002**	**26.5 mediation**

*Note:* Mediation structural equation model was adjusted for sex, family history of hypertension/diabetes/high cholesterol/vascular disease, socio‐economic status and time‐varying covariates measured at both baseline and follow‐up such as age, heart rate, smoking status, light physical activity and moderate‐to‐vigorous physical activity, with additional adjustments for fat mass, lean mass, insulin resistance, high‐sensitivity C‐reactive protein, high‐density lipoprotein cholesterol, low‐density lipoprotein cholesterol or triglyceride depending on the mediator. *β* is standardized regression coefficient. *P*‐values < 0.05 of indirect effects are considered statistically significant and are bolded. When the magnitude of the longitudinal association between the predictor and outcome is increased upon inclusion of a third variable, a suppression is confirmed. However, when decreased, it is mediation.

Cumulative HDL‐c, LDL‐c, triglyceride, hsCRP and insulin resistance had no statistically significant mediating effect on the inverse associations of cumulative LPA with cumulative systolic and diastolic BP (Table 5). Cumulative total fat mass partially mediated (5.4%–6.6%) the inverse associations of cumulative LPA with cumulative systolic and diastolic BP (Table 5). Cumulative lean mass partly mediated (18%–20%) the inverse associations of cumulative LPA with cumulative systolic and diastolic BP (Table 5).

**TABLE 5:** Mediating or suppressing role of cumulative fat mass, lean mass and fasting insulin resistance, lipids and inflammation on the longitudinal associations of cumulative light physical activity and blood pressure progression from ages 11 through 24 years of 2513 participants.

	Cumulative systolic blood pressure from ages 11 to 24 years						
Cumulative light physical activity	Total effect		Direct effect		Indirect effect		Mediation or suppression (%)
Mediators	*β (95% CI)*	*p*	*β (95% CI)*	*p*	*β (95% CI)*	*p*	
HDL	−0.319 (−0.339 to −0.297)	0.003	−0.315 (−0.333 to −0.293)	0.004	−0.004 (−0.013–0.004)	0.332	1.3
LDL	−0.246 (−0.266 to −0.225)	0.002	−0.245 (−0.265 to −0.224)	0.002	−0.001 (−0.003–0.000)	0.116	0.4
Triglyceride	−0.237 (−0.258 to −0.216)	0.003	−0.236 (−0.257 to −0.215)	0.002	−0.002 (−0.006–0.002)	0.334	0.8
Insulin resistance	−0.251 (−0.271 to −0.229)	0.003	−0.253 (−0.273 to −0.231)	0.003	0.001 (−0.001–0.004)	0.216	0.4
High‐sensitivity CRP	−0.235 (−0.257 to −0.212)	0.003	−0.233 (−0.255 to −0.211)	0.003	−0.002 (−0.006–0.002)	0.326	0.6
Fat mass	−0.182 (−0.206 to −0.158)	0.002	−0.169 (−0.194 to −0.145)	0.002	−0.012 (−0.019 to −0.005)	**0.002**	**6.6 mediation**
Lean mass	0.160 (0.125–0.190)	0.003	0.129 (0.105–0.153)	0.002	0.032 (0.008–0.054)	**0.011**	**20.0 mediation**

Cumulative light physical activity	Cumulative diastolic blood pressure from ages 11 to 24 years						
	Total effect		Direct effect		Indirect effect		Mediation or suppression (%)
Mediators	*β (95% CI)*	*p*	*β (95% CI)*	*p*	*β (95% CI)*	*p*	
HDL	−0.352 (−0.371 to −0.331)	0.004	−0.349 (−0.369 to −0.328)	0.004	−0.002 (−0.008–0.002)	0.324	0.6
LDL	−0.302 (−0.321 to −0.282)	0.003	−0.300 (−0.319 to −0.280)	0.003	−0.002 (−0.004–0.001)	0.185	0.7
Triglyceride	−0.300 (−0.320 to −0.280)	0.003	−0.298 (−0.317 to −0.277)	0.003	−0.002 (−0.006–0.002)	0.334	0.7
Insulin resistance	−0.310 (−0.329 to −0.291)	0.003	−0.317 (−0.335 to −0.297)	0.004	0.007 (0.003–0.012)	**0.002**	**2.3 mediation**
High‐sensitivity CRP	−0.291 (−0.311 to −0.270)	0.003	−0.289 (−0.310 to −0.268)	0.003	−0.003 (−0.008–0.003)	0.324	1.1
Fat mass	−0.240 (−0.261 to −0.214)	0.003	−0.227 (−0.248 to −0.201)	0.003	−0.013 (−0.021 to −0.006)	**0.002**	**5.4 mediation**
Lean mass	−0.095 (−0.122 to −0.064)	0.003	−0.112 (−0.135 to −0.084)	0.003	0.017 (0.004–0.029)	**0.011**	**17.9 mediation**

*Note:* Mediation structural equation model was adjusted for sex, family history of hypertension/diabetes/high cholesterol/vascular disease, socio‐economic status and time‐varying covariates measured at both baseline and follow‐up such as age, heart rate, smoking status, sedentary time and moderate‐to‐vigorous physical activity, with additional adjustments for fat mass, lean mass, insulin resistance, high‐sensitivity C‐reactive protein, high‐density lipoprotein cholesterol, low‐density lipoprotein cholesterol or triglyceride depending on the mediator. *β* is standardized regression coefficient.  *P*‐values < 0.05 of indirect effects are considered statistically significant and are bolded. When the magnitude of the longitudinal association between the predictor and outcome is increased upon inclusion of a third variable, a suppression is confirmed. However, when decreased it is mediation.

Cumulative triglyceride, hsCRP, insulin resistance and lean mass had no statistically significant mediating effect on the inverse associations of cumulative MVPA with cumulative systolic and diastolic BP (Table 6). Cumulative LDL‐c partially mediated (1.5%–3.3%) the inverse associations of cumulative MVPA with cumulative systolic and diastolic BP (Table 6). Cumulative HDL‐c partially mediated (6.2%) the inverse associations of cumulative MVPA with cumulative systolic BP but not diastolic BP. Cumulative total fat mass partially mediated (19%–27%) the inverse associations of cumulative MVPA with cumulative systolic and diastolic BP (Table 6 and Figure 1).

**TABLE 6:** Mediating or suppressing role of cumulative fat mass, lean mass, and fasting insulin resistance, lipids and inflammation on the longitudinal associations of cumulative moderate‐to‐vigorous physical activity and blood pressure progression from ages 11 through 24 years of 2513 participants.

Cumulative moderate‐to‐vigorous physical activity	Cumulative systolic blood pressure from ages 11 to 24 years						
	Total effect		Direct effect		Indirect effect		Mediation or Suppression (%)
Mediators	*β (95% CI)*	*p*	*β (95% CI)*	*p*	*β (95% CI)*	*p*	
HDL	−0.145 (−0.168 to −0.122)	0.002	−0.136 (−0.159 to −0.113)	0.003	−0.009 (−0.012 to −0.006)	**0.001**	**6.2 mediation**
LDL	−0.138 (−0.161 to −0.115)	0.003	−0.136 (−0.158 to −0.112)	0.003	−0.002 (−0.005 to −0.001)	**0.011**	**1.5 mediation**
Triglyceride	−0.136 (−0.159 to −0.114)	0.002	−0.136 (−0.159 to −0.113)	0.002	0.000 (−0.002–0.003)	0.947	0
Insulin resistance	−0.145 (−0.168 to −0.122)	0.002	−0.145 (−0.168 to −0.122)	0.002	0.000 (−0.001–0.000)	0.405	0
High‐sensitivity CRP	−0.130 (−0.153 to −0.107)	0.002	−0.130 (−0.153 to −0.107)	0.002	0.000 (−0.003–0.003)	0.939	0
Fat mass	‐0.107 (−0.129 to −0.084)	0.003	−0.086 (−0.108 to −0.063)	0.002	−0.020 (−0.027 to −0.014)	**0.003**	**18.7 mediation**
Lean mass	−0.173 (−0.197 to −0.148)	0.003	−0.176 (−0.196 to −0.154)	0.003	0.003 (−0.005–0.013)	0.472	1.7

Cumulative moderate‐to‐vigorous physical activity	Cumulative diastolic blood pressure from ages 11 to 24 years						
	Total effect		Direct effect		Indirect effect		Mediation or suppression (%)
Mediators	*β (95% CI)*	*p*	*β (95% CI)*	*p*	*β (95% CI)*	*p*	
HDL	−0.131 (−0.154 to −0.109)	0.003	−0.131 (−0.154 to −0.108)	0.002	−0.001 (−0.002–0.001)	0.393	0.8
LDL	−0.123 (−0.146 to −0.101)	0.002	−0.119 (−0.142 to −0.096)	0.002	−0.004 (−0.007 to −0.001)	0.011	**3.3 mediation**
Triglyceride	−0.123 (−0.146 to −0.101)	0.003	−0.123 (−0.147 to −0.101)	0.002	0.000 (−0.003–0.003)	0.947	0
Insulin resistance	−0.127 (−0.150 to −0.104)	0.003	−0.127 (−0.150 to −0.105)	0.002	0.000 (−0.001–0.001)	0.553	0
High‐sensitivity CRP	−0.113 (−0.136 to −0.091)	0.002	−0.112 (−0.136 to −0.091)	0.002	0.000 (−0.004–0.004)	0.943	0
Fat mass	−0.088 (−0.110 to −0.066)	0.002	−0.063 (−0.086 to −0.041)	0.002	−0.024 (−0.031 to −0.017)	**0.003**	**27.3 mediation**
Lean mass	−0.153 (−0.178 to −0.126)	0.003	−0.155 (−0.178 to −0.129)	0.003	0.002 (−0.003–0.009)	0.466	1.3

*Note:* Mediation structural equation model was adjusted for sex, family history of hypertension/diabetes/high cholesterol/vascular disease, socio‐economic status and time‐varying covariates measured at both baseline and follow‐up such as age, heart rate, smoking status, sedentary time and light physical activity with additional adjustments for fat mass, lean mass, insulin resistance, high‐sensitivity C‐reactive protein, high‐density lipoprotein cholesterol, low‐density lipoprotein cholesterol or triglyceride depending on the mediator. *β* is standardized regression co‐efficient. *P*‐values < 0.05 of indirect effects are considered statistically significant and are bolded. When the magnitude of the longitudinal association between the predictor and outcome is increased upon inclusion of a third variable, a suppression is confirmed. However, when decreased it is mediation.

## 4 Discussion

In the largest and longest follow‐up study of children with objectively measured movement behaviours and repeated BP measures, it was observed that ST may be an independent and potential causal risk factor for increased systolic and diastolic BP from childhood through young adulthood and LPA may be the ideal intervention to prevent and reverse increased BP progression. In the confounding‐based longitudinal analyses, cumulative ST was independently and directly associated with increased systolic and diastolic BP over 13 years of follow‐up, whereas cumulative LPA was inversely associated with BP progression. Cumulative MVPA was not associated with decreased BP due to lean mass confounding effects. Next, theoretically replacing time spent sedentary with an equal amount of time spent in LPA but not MVPA significantly lowered BP during growth from childhood through young adulthood. Lastly, in the mediation analyses, it was observed that lean mass mediated the longitudinal relationships of cumulative ST or LPA with BP, whereas fat mass mediated the longitudinal relationships of MVPA with BP. Lipids and inflammation were not significant mediators in the longitudinal associations of movement behaviour with BP.

### 4.1 ST and BP

The consistency in the positive and independent longitudinal associations of cumulatively increased ST and progressive increase in both systolic and diastolic BP independent of LPA and MVPA and irrespective of model strategy suggests that ST may potentially cause an adverse increase in BP during growth from childhood through young adulthood [8, 9, 13, 31]. A large‐scale cross‐sectional study reported that reallocating 10 min of ST with either LPA or MVPA was associated with lower systolic BP 0.03 and 0.08 mmHg, respectively, among 8100 adolescents average aged 11.6 years, but not among 3544 children aged 8.1 years or 1865 adolescents aged 16.2 years [32].

In the present longitudinal study, ST steadily increased from ~6 h/day in childhood to ~9 h/day by young adulthood. Among adults, > 10 h/day of ST was associated with an increased risk of incident cardiovascular events [8, 33]. The cumulative increase in ST from childhood to young adulthood was associated with a cumulative +4 mmHg increase in systolic BP in the fully adjusted model. It is of public health and clinical significance that a longitudinal replacement of 10 min spent in ST with equal time in LPA during childhood, adolescence and young adulthood theoretically decreased BP by approximately −3 mmHg systolic BP and −2 mmHg diastolic BP. A large‐scale meta‐analysis of randomized controlled trials concluded that a 5 mmHg reduction of systolic BP reduced the risk of major cardiovascular events by ~10% [34].

It is noteworthy that tripling the theoretical replacement time from 10 to 30 min did not proportionally correspond to a threefold decrease in BP; rather, the BP‐lowering effect of the 10 min of LPA replacement was reduced by ~70% when 10‐min LPA replacement was increased to 30 min. A plausible explanation might be that the cardiovascular system physiologically adapts to prolonged exercise [35, 36, 37]. Recently, we reported that increasing time spent in PA yields more metabolic (reduced fat, lipids and inflammation) and liver health benefits but the benefit on cardiovascular system plateaus [10, 35, 38, 39, 40, 41, 42]. Prolonged exercise increases the potential for arterial stiffening due to an increase in smooth muscle deposit in the vascular wall and excessive arterial stiffness may increase BP [29, 35]. In addition, exercise slightly increases left ventricular mass in children and adolescents [27, 36, 37]. A 10 mmHg increase in diastolic BP may likely be physiologic if the baseline is low, for example, 50 mmHg, but a 10 mmHg increase from a baseline value of 70 mmHg may not be physiologic. The average diastolic BP in this population at age 24 years is 67 mmHg. This observation fills several identified knowledge gaps and is important for planning general paediatric population public health interventions in preventing and lowering BP because the WHO recently estimated that half a billion new cases of physical inactivity‐related non‐communicable diseases would occur between 2020 and 2030 and 47% of those cases would result from hypertension [1, 7, 8, 9, 13, 14, 20]. The mediation analyses showed that lean mass had almost 93% mediation effect in the positive associations of ST with systolic BP. This observation contradicts previous studies that have concluded that increased body mass index was the mediator between increased ST and BP [7, 8, 9, 43]. The availability of gold‐standard dual‐energy x‐ray absorptiometry measures of body composition from childhood through young adulthood improves our understanding that fat mass was not a statistically significant mediator between ST and BP, but rather lean mass. Emerging longitudinal evidence supports lean mass as a driver of cardiovascular adaptation and remodelling during growth from childhood through young adulthood [28, 35, 41, 43, 44, 45]. Paradoxically, cumulative insulin resistance appears to partly suppress the positive associations of ST with BP. We recently showed that the physiologic decline in insulin resistance during growth from mid‐adolescence through young adulthood significantly attenuated worsening vascular stiffness [46]. It has been reported that physical inactivity lasting 6 days reduced insulin action through the reduction in Glut‐4 levels in endurance‐trained runners' skeletal muscle [3, 4, 9]. ST has been associated with increased endoplasmic reticulum oxidative stress, inflammation and mitochondrial dysfunction, which could result in beta cell insufficiency and decreased insulin secretion [4, 9, 47]. It thus seems that optimal insulin resistance levels in relatively healthy young populations may offer mild protection against the deleterious effect of ST on BP as the magnitude of the suppression effect of insulin resistance on the relationship between ST and BP was ~7%. Although ST has been associated with increased lipids and inflammation, neither lipids nor inflammation had any mediating or suppressing role in the relationship of cumulative ST with BP [10, 39, 40].

### 4.2 LPA and BP

There is a substantial lack of longitudinal evidence regarding accelerometer‐measured LPA in relation to BP in the paediatric population, especially for those who lack motivation or are unable to achieve the recommended guideline of at least 60 min/day of MVPA [1, 13, 20, 31, 32]. In the present longitudinal study, cumulative LPA was consistently associated with decreased systolic BP from childhood through young adulthood, irrespective of adjustment or modelling strategy. A potential causal effect of the BP‐lowering effect of LPA seems biologically plausible and could be adopted in clinical BP and hypertension guidelines. The potential causal presupposition is based on consistency, exchangeability, dose–response, treatment effect, biological plausibility and temporality of the relationship between LPA and lower BP. A recent study among adolescents suggests that engaging in LPA may be associated with better cardiac health than engaging in MVPA [27]. Among adults, LPA has been associated with reduced risk of all‐cause mortality and Parkinson's diseases [48, 49]. Although cumulative LPA was associated with lower BP in females, it was associated with higher BP in males. This sex disparity in findings may be related to males accumulating more LPA and MVPA and having a significantly higher lean mass than females [38].

The cumulative increase in total body fat mass from childhood through young adulthood partially mediated the inverse associations of cumulative LPA with BP with an approximately 5%–7% mediation effect. Thus, engaging in LPA while increasing body fat may slightly reduce the health benefit of LPA in lowering BP, inflammation and dyslipidaemia as previously reported [10, 40, 50]. Lifestyle modification that includes both improved movement behaviour and diet may be a more comprehensive strategy for decreasing BP in the paediatric population [20]. Over the 13‐year follow‐up, participants accumulated an average of 5 h (305 min)/day of LPA. This LPA duration might be required to significantly lower systolic BP, because 305 min/day of LPA from ages 11 through 24 years was associated with an approximately −2 mmHg decrease in cumulative systolic BP over the 13‐year follow‐up. These independent BP‐lowering characteristics of LPA may have clinical and public health significance [10, 14, 34, 38, 40]. The prevalence of elevated BP and hypertension in youth is 6%–12%, and paediatricians may integrate questions on sedentary behaviour in the evaluation of major risk factors for developing arterial hypertension in youth and recommend LPA as a better preventive or treatment strategy [15]. Nonetheless, future randomized clinical trials in the general paediatric population and mechanistic studies are warranted to examine LPA's effect, particularly a long LPA duration of over 5 h/day in reducing BP [1].

### 4.3 MVPA and BP

To accumulate at least 60 min/day of MVPA on average in children and adolescents was recently recommended by the WHO; however, the benefit of MVPA on lowering BP has not been fully established in the paediatric population due to null results from several randomized clinical trials [1, 12, 13, 20]. In the current study, neither persistent ≥ 60 min/day of MVPA as a categorical variable nor MVPA as a continuous variable throughout the 13‐year follow‐up period was associated with decreased systolic BP. Paradoxically, cumulative MVPA and persistent ≥ 60 min/day of MVPA were independently associated with increased diastolic BP from childhood through young adulthood in the total cohort and among males. Higher MVPA has been associated with higher left ventricular mass in adolescents, which could also relate to an increasing BP [27, 36]. It was further observed that when lean mass was excluded from the model, both cumulative MVPA and persistent ≥ 60 min/day were inversely associated with systolic BP but not diastolic BP. This result suggests a strong confounding effect of lean mass in the relationship between MVPA and BP and could explain why several randomized clinical trials have been unsuccessful in lowering BP [11, 12]. A recent randomized clinical trial reported baseline values of 18‐ to 35‐year‐old adults with awake 24‐h BP 115/75–159/99 mmHg. After the trial, there were no group differences in awake systolic (0 mmHg [95% CI, −2.9–2.8]; *p* = 0.98) or awake diastolic ambulatory blood pressure (0.6 mmHg [95% CI, −1.4–2.6]; *p* = 0.58) [12]. MVPA increases lean mass, and lean mass has been associated with a significant increase in BP [28, 38].

In the mediation analysis, it was observed that the potential inverse association of cumulative MVPA with BP was approximately 20%–30% mediated by total body fat mass, but no significant mediating effect of lean mass was observed. Taken together, the MVPA effect on BP appeared to be strongly dampened by the mediating and confounding effect of body composition. It is noteworthy that the theoretical replacement of either 10 or 30 min of ST with an equal time of MVPA did not significantly lower BP from childhood through young adulthood. Importantly, lean mass also confounded the theoretical replacement of minutes spent in ST with MVPA. Overall, cumulative time spent in MVPA from childhood to young adulthood was associated with no increase or decrease (±0 mmHg) in systolic BP in the fully adjusted model. Future clinical trials, guidelines and public health messages may focus on promoting LPA for lowering BP in the young population rather than MVPA [13, 19].

### 4.4 Strength and Limitations

The ALSPAC dataset provides an extensive array of gold‐standard and repeated measures of movement behaviours, BP, body composition and covariates throughout the follow‐up period in a large paediatric population. Using advanced statistical models, we tested the likelihood of theoretical activity displacement, potential causal explanatory pathway and consistency of the longitudinal findings for the first time in a large paediatric population. Our findings aptly fill several knowledge gaps recently identified in movement behaviour research and would be useful in updating future PA guidelines and WHO reports [1, 7, 9, 13, 14, 20]. On the other hand, the present study had some limitations. Our participants were mostly White; therefore, we are unable to generalize our findings to other racial and ethnic groups. Moreover, as with all observational studies, residual biases due to unmeasured confounders may distort observed associations such as the unavailability of quantitative sleep variables, parental PA data and dietary records. Nonetheless, adjustment of energy intake during adolescence did not alter the results. Participants who spent more time sedentary may have a less healthy (high‐salted) diet than those with a physically active lifestyle, which might impact BP. Also, cohort attrition could lead to bias; however, participants who lacked certain movement behaviour and BP variables had similar characteristics to those included in the analyses. Moreover, conducting the analysis with stricter inclusion criteria of having at least two time‐point valid measures of movement behaviour and complete BP measures across all time points resulting in approximately 1000 fewer participants did not alter the results. Clinic BP measurement during a single research visit is limited in diagnosing hypertension. Unfortunately, there were no data on ambulatory BP measures; thus, future research may examine how movement behaviours longitudinally associates with ambulatory BP and masked hypertension in the young population. There could be residual bias due to different BP and accelerometer devices used during the 13‐year follow‐up period, but these devices have shown excellent agreements (*r* > 0.90).

## 5 Conclusion

Over a 13‐year follow‐up period from childhood through young adulthood, cumulative ST was consistently associated with increased systolic (+4 mmHg) and diastolic BP, whereas cumulative LPA was associated with decreased systolic BP (−2 mmHg). Both MVPA as a continuous variable and persistent exposure to ≥ 60 min/day of MVPA as a categorical variable were not associated with decreased systolic BP (±0 mmHg) but paradoxically associated with increased diastolic BP over the 13‐year observation period. Lean mass significantly confounded the relationships of MVPA with BP. Longitudinal isotemporal replacement of 10 min of time spent in ST with equal time in LPA during childhood, adolescence and young adulthood theoretically decreased cumulative systolic BP by approximately −3 mmHg and diastolic BP by −2 mmHg, which is clinically significant. Future guidelines could emphasize the importance of early LPA intervention from childhood for the prevention of elevated BP and hypertension.

## Author Contributions

Andrew O. Agbaje had full access to all the data in the study and take responsibility for the integrity of the data and the accuracy of the data analysis. *Concept and design*: Andrew O. Agbaje. *Acquisition, analysis and interpretation of data:* Andrew O. Agbaje. *Drafting of the manuscript*: Andrew O. Agbaje. *Critical revision of the manuscript for important intellectual content:* Andrew O. Agbaje. *Statistical analysis:* Andrew O. Agbaje. *Obtained funding:* Andrew O. Agbaje. This publication is the work of the author, and Andrew O. Agbaje will serve as guarantor for the contents of this paper.

## Disclosure

The author has nothing to report.

## Conflicts of Interest

The author declares no conflicts of interest.

## Supporting information


**Figure S1.** Flowchart of cohort participants. LPA, light physical activity; MVPA, moderate‐to‐vigorous physical activity; ST, sedentary time. Altogether 5217 participants attended the age 17‐year clinic visits between October 2008 and December 2010 of which 4953 participants had valid blood sample measures.
**Figure S2.** Directed Acyclic Graph on the potential causal relationship between physical activity and blood pressure, illustrating mediators (golden colour), confounders (green colour) and unmeasured confounders (red colour). CRP, high sensitivity C‐reactive protein; FHx, family history of cardiovascular, cholesterol, diabetes, and hypertensive diseases; FM, fat mass; HDL, high‐density lipoprotein cholesterol; HR, heart rate; Glu, glucose or insulin; LDL, low‐density lipoprotein cholesterol; LM, lean mass; SMO, smoking; ST, sedentary time; Trig, triglyceride; U, Unmeasured covariates.
**Table S1.** Characteristics of participants excluded from the study based on lack of complete three‐time‐point blood pressure measurement at age 11‐, 15‐, and 24‐years clinic visit using the age 15‐year clinic visit profile.
**Table S2.** Sex‐specific longitudinal associations of cumulative sedentary time and physical activity with blood pressure progression from ages 11 through 24 years of 2513 participants who had at least one‐time point movement behaviour measure and complete three‐time point blood pressure measure.
**Table S3.** Longitudinal associations of cumulative sedentary time and physical activity with blood pressure progression from ages 11 through 24 years among participants with at least two‐time point movement behaviour measure and complete three‐time point blood pressure measure.
**Table S4.** Complete case analysis of longitudinal associations of cumulative sedentary time and physical activity with blood pressure progression from ages 11 through 24 years among participants with at least one‐time point movement behaviour measure and complete three‐time point blood pressure measure.
**Table S5.** Compositional data analysis of the longitudinal associations of cumulative sedentary time and physical activity with blood pressure progression from ages 11 through 24 years among participants with at least one‐time point movement behaviour and blood pressure measure.

## Data Availability

The informed consent obtained from ALSPAC participants does not allow the data to be made freely available through any third‐party maintained public repository. However, data used for this submission can be made available on request to the ALSPAC Executive. The ALSPAC data management plan describes in detail the policy regarding data sharing, which is through a system of managed open access. Full instructions for applying for data access can be found here: http://www.bristol.ac.uk/alspac/researchers/access/. The ALSPAC study website contains details of all the data that are available (http://www.bristol.ac.uk/alspac/researchers/our‐data/).
