# Supplementary material for: Lean Mass Longitudinally Confounds Sedentary Time and Physical Activity With Blood Pressure Progression in 2513 Children
Source: J Cachexia Sarcopenia Muscle. 2024 Nov 13;15(6):2826–41. doi: 10.1002/jcsm.13639 (PMC11634498; doi:10.1002/jcsm.13639)
Supplement: Supplementary file 1 — Figure S1. Flowchart of cohort participants. LPA, light physical activity; MVPA, moderate‐to‐vigorous physical activity; ST, sedentary time. Altogether 5217 participants attended the age 17‐year clinic visits between October 2008 and December 2010 of which 4953 participants had valid blood sample measures. Figure S2. Directed Acyclic Graph on the potential causal relationship between physical activity and blood pressure, illustrating mediators (golden colour), confounders (green colour) and unmeasured confounders (red colour). CRP, high sensitivity C‐reactive protein; FHx, family history of cardiovascular, cholesterol, diabetes, and hypertensive diseases; FM, fat mass; HDL, high‐density lipoprotein cholesterol; HR, heart rate; Glu, glucose or insulin; LDL, low‐density lipoprotein cholesterol; LM, lean mass; SMO, smoking; ST, sedentary time; Trig, triglyceride; U, Unmeasured covariates. Table S1. Characteristics of participants excluded from the study based on lack of complete three‐time‐point blood pressure measurement at age 11‐, 15‐, and 24‐years clinic visit using the age 15‐year clinic visit profile. Table S2. Sex‐specific longitudinal associations of cumulative sedentary time and physical activity with blood pressure progression from ages 11 through 24 years of 2513 participants who had at least one‐time point movement behaviour measure and complete three‐time point blood pressure measure. Table S3. Longitudinal associations of cumulative sedentary time and physical activity with blood pressure progression from ages 11 through 24 years among participants with at least two‐time point movement behaviour measure and complete three‐time point blood pressure measure. Table S4. Complete case analysis of longitudinal associations of cumulative sedentary time and physical activity with blood pressure progression from ages 11 through 24 years among participants with at least one‐time point movement behaviour measure and complete three‐time point blood pressure measure. [file JCSM-15-2826-s001.pdf]

Lean Mass Longitudinally Confounds Sedentary Time and Physical Activity with Blood Pressure Progression in 2513 Children

Andrew O. Agbaje MD, MPH, PhD, FESC, FAHA[1,2]

[1]*Institute of Public Health and Clinical Nutrition, School of Medicine, Faculty of Health Sciences, University of Eastern Finland, Kuopio, Finland.*

[2]*Children's Health and Exercise Research Centre, Department of Public Health and Sports Sciences, Faculty of Health and Life Sciences, University of Exeter, Exeter, United Kingdom.*

.

Address correspondence to:
Andrew O. Agbaje, MD, MPH, PhD, FESC, FAHA, FNYAM Cert. Clinical Research *(Harvard)*
Professor (associate) of Clinical Epidemiology and Child Health.
Institute of Public Health and Clinical Nutrition, School of Medicine, Faculty of Health Sciences, University of Eastern Finland, Kuopio Campus.
Address: Yliopistonranta 8, P.O. Box 1627, 70211 Kuopio, Finland
E-mail: andrew.agbaje@uef.fi

Unabridged Method

Methods

Study cohort

Data were from the ALSPAC birth cohort, which investigates factors that influence childhood development and growth. Pregnant women resident in Avon, UK with expected dates of delivery between 1st April 1991 and 31st December 1992 were invited to take part in the study. 20,248 pregnancies have been identified as being eligible and the initial number of pregnancies enrolled was 14,541. Of the initial pregnancies, there was a total of 14,676 foetuses, resulting in 14,062 live births and 13,988 children who were alive at 1 year of age. When the oldest children were approximately 7 years of age, an attempt was made to bolster the initial sample with eligible cases who had failed to join the study originally. As a result, when considering variables collected from the age of seven onwards (and potentially abstracted from obstetric notes) there are data available for more than the 14,541 pregnancies mentioned above. The number of new pregnancies not in the initial sample (known as Phase I enrolment) that are currently represented in the released data and reflecting enrolment status at the age of 24 is 906, resulting in an additional 913 children being enrolled (456, 262 and 195 recruited during Phases II, III and IV respectively). The total sample size for analyses using any data collected after the age of seven is therefore 15,447 pregnancies, resulting in 15,658 foetuses. Of these 14,901 children were alive at 1 year of age. Regular clinic visits of the children commenced at 7 years of age and are still ongoing into adulthood. Study data at 24 years of age were collected and managed using REDCap electronic data capture tools.[1] In this study, 2513 participants with complete BP measures at age 11, 15, and 24 years clinic visits, and at least one time-point valid ST, LPA, and MVPA measurements at either age 11, 15, or 24 years clinic visit were eligible for analyses (Supplemental Figure 1). The excluded participants who had at least one timepoint measure of ST and PA and incomplete BP measures during the 13-year-long follow-up study had similar characteristics with those included in the study (Supplemental

Table 1). To increase the robustness of the results, we applied stricter inclusion criteria of at least two-time point valid measures of ST and PA with complete BP measures during the 13-year follow-up, which resulted in 1556 participants. Ethical approval for the study was obtained from the ALSPAC Ethics and Law Committee and the Local Research Ethics Committees. Informed consent for the use of data collected via questionnaires and clinics was obtained from participants following the recommendations of the ALSPAC Ethics and Law Committee at the time.[2–4] Consent for biological samples has been collected in accordance with the Human Tissue Act (2004). Please note that the study website contains details of all the data that is available through a fully searchable data dictionary and variable search tool (http://www.bristol.ac.uk/alspac/researchers/our-data/).

Exposures: Sedentary time and physical activity assessment

ST, LPA, and MVPA were assessed with ActiGraph™ (LLC, Fort Walton Beach, FL, USA) accelerometer worn on the waist for 7 consecutive days at 11- and 15-year clinic visits whereas at 24 years movement behaviour was assessed using ActiGraph GT3X+ accelerometer device worn for four consecutive days.[5–8] There is a strong absolute agreement between the Actigraph™ models (intraclass correlation coefficient 0.99 (95% CI = 0.98–0.99) thus making it acceptable to use different models within a study.[9] A valid day was defined as providing data for at least 10 hours per day (excluding sequences of 10 or more minutes with consecutive zero counts) and children were only included in the analyses if they provided at least 3 valid days of recording. The devices capture movement in terms of acceleration as a combined function of frequency and intensity. Data are recorded as counts that result from summing postfiltered accelerometer values (raw data at 30 Hz) into 60 seconds epoch units. Data were processed using Kinesoft software, version 3.3.75 (Kinesoft), according to established protocol.[10] Activity counts per minute threshold validated in children and adolescents were used to calculate the amount of time spent; MVPA, >2296 counts per minute (cpm); for LPA, 100 – 2296 cpm; and for ST, 0 – <100 cpm at ages 11 and 15 years using the Evenson cutpoint whereas, at the 24-year assessment, the

2020 cpm Troiano cut point was used.[10–12] The Evenson cutpoint used in stratifying activity threshold has shown the best overall performance across all intensity levels and was suggested as the most appropriate cut point for youth.[11,12] Pearson bi-variate correlations (r^2) between cumulative 11, 15, and 24 years ST and LPA are (-0.71), ST and MVPA (-0.30), LPA and MVPA (0.17), p-value <0.001 for all. For each age clinic visit, at age 11 years, the correlations between ST and LPA are (-0.58), ST and MVPA (-0.33), LPA and MVPA (0.15), p-value <0.001 for all. At age 15 years clinic visit, the correlations between ST and LPA are (-0.48), ST and MVPA (-0.31), LPA and MVPA (0.27), p-value <0.001 for all. At age 24 years clinic visit, the correlations between ST and LPA are (-0.22), p = 0.009, ST and MVPA (-0.19), p = 0.002, LPA and MVPA (0.13), p-value <0.001 for all. These correlation matrices are similar to the report of meta-analysis of cross-sectional accelerometer studies.[13] MVPA was classified as <40 minutes/day as low (reference), 40 – <60 minutes/day as moderate, and ≥60 minutes/day as high. The 40 – <60 minutes/day of MVPA was based on the lowest tertile cutpoint (39.9 minutes) for MVPA in the total population. The cut point between the middle and highest MVPA tertile was 60.7 mins/day in line with the current PA guideline.[14]

Outcomes: Blood Pressure Measures

At age 11- and 15-years clinic visit, BP was measured using a Dinamap 9301 Vital Signs monitor. The child was first given a simple explanation of what would happen in the session using the analogy of an inflating balloon to explain the action of the cuff. Two cuffs were used depending on the size of the child's upper arm circumference (ideally the right arm was used): If < 23cm a small adult-size cuff was used and if ≥ 23cm an adult cuff was used. Two readings of systolic and diastolic BP were recorded and the mean of each was calculated for analysis. If the child's BP was 140/90mmHg or more the parents were given a letter to take to their physician.[15] At age 24-year clinic visit, BP readings were taken using an Omron M6 upper arm BP. Participants were asked to sit and rest for two minutes prior to taking the first measurement.[15–18]

Confounders and covariates: Anthropometry, body composition, cardiometabolic, socioeconomic, and lifestyle factors

Anthropometry (height and weight) of participants at ages 11, 15, and 24 years were assessed in line with standard protocols, and body mass index was computed as weight in kilograms per height in meters squared.[15,17] Body composition (total body fat mass and total body lean mass) was assessed using dual energy Xray absorptiometry scanner at 11, 15, and 24 years clinic visits as previously described.[15,17,19] Heart rate was measured with semi-automated digital monitors at ages 11, 15, and 24 years as previously detailed.[15,17] Using standard protocols, fasting blood samples at ages 15, 17, and 24 years were collected, spun, and frozen at –80 °C, and a detailed assessment of fasting glucose, insulin, high-sensitivity C-reactive protein (hsCRP), low-density lipoprotein cholesterol (LDL-c), high-density lipoprotein cholesterol (HDL-c), and triglycerides has been reported (coefficient of variation was <5%).[15,17,19] Fasting insulin was assessed using an ultrasensitive automated microparticle enzyme immunoassay (ELISA, Mercodia, DSL, London, UK), ALSPAC (RRID:SCR_007260).[20] The homeostatic model assessment of insulin resistance (HOMA-IR) was computed from (fasting insulin × fasting glucose/22.5). At the 17-year clinic visit, participants were briefly asked about their personal and family (mother, father, and siblings) medical history such as a history of hypertension, diabetes, high cholesterol, and vascular disease. All participants had attained puberty at 17 years clinic visit using a time (years) to age at peak height velocity objective assessment derived from Superimposition by Translation And Rotation mixed-effects growth curve analysis.[15,21]

The participant's mother's socioeconomic status was grouped according to the 1991 British Office of Population and Census Statistics classification.[22] Questionnaires to assess smoking behaviour were administered at the 13, 15, and 24-year clinic visits. A specific question regarding whether participants smoked in the last 30 days was used as an indicator of current smoking status.

Handling of missing covariates and multiple imputations

Eligible sample size varied by covariates and exclusions via listwise deletion of missing values ranged from 0.2 to 52.6 percent for covariates at either 11, 15, or 24-year clinic visits. We restricted study participants to those who had complete outcome variables at all study time points at

ages 11, 15, and 24 years. We conducted a Little's missing completely at random (MCAR) test to ascertain data missingness.[23] Little's MCAR test: Chi-Square = 5421.59, degree of freedom = 4684, p-value <0.0001, made us conclude that the variables were not missing completely at random. Regression-modeled multiple imputations were conducted using SPSS version 27 (IBM Corp, Armonk, NY, USA). The observed minimum and maximum values were constraints for the imputation process and 20 cycles of imputation with 10 iterations resulted in 20 imputed data sets. The multiple imputation module in SPSS pooled the results from these imputed data. In line with previous evidence,[17] the percentage of missing values would be sufficiently addressed with 20 imputations: the variable with the highest missing value (52.6%, maternal socioeconomic status) had an estimate that was 98% efficient after 20 imputations (computed using Rubin's formula).[23] The distributions of imputed variables had the same pattern as in the observed data as evidenced in a histogram normality plot. Where multiple imputations have been conducted, presenting imputed results is preferred to presenting non-imputed results.[24]

Statistical analysis

Cohort descriptive characteristics were summarized as means and standard deviation, medians and interquartile ranges, or frequencies and percentages. We explored sex differences using independent t-tests, Mann Whitney-U tests, or Chi-square tests for normally distributed, skewed, or dichotomous variables, respectively. Multicategory variables were analysed using a one-way analysis of variance. Normality was assessed by histogram curve, quantile-quantile plot, and Kolmogorov-Smirnov tests with p-valve >0.05. We conducted a logarithmic transformation of skewed variables and confirmed normality prior to further analysis (mediation analysis).

Analyses of longitudinal associations (single and partition models)

We examined the separate longitudinal associations of each of the 13-year ST, LPA, and MVPA progression (11 through 24 years) with each of systolic and diastolic BP measured at ages 11, 15, and 24 years using generalized linear mixed-effect models (GLMM). The GLMM is robust for

handling highly correlated variables such as ST and LPA.[25] The optimal model with the lowest Bayesian Information Criteria was one with sex as a main effect, a random intercept modeled for the participants to account for within-individual correlations. Whilst the GLMM is robust for handling missing at random predictor and covariate data, we elected to additionally conduct 20 cycles of multiple imputations to account for missing data. The GLMM accounted for baseline ST, LPA, MVPA predictors, BP outcomes, and covariates and their repeated measures. For ST, LPA, and MVPA continuous variable analyses, Model 1 was unadjusted. Model 2 was adjusted for sex, and other time-varying covariates measured at both baseline and follow-up such as age, LDL-c, triglyceride, hsCRP, HDL-c, heart rate, glucose, insulin, smoking status, family history of hypertension/diabetes/high cholesterol/vascular disease, socioeconomic status, fat mass, and lean mass. Model 3 was an additional adjustment for either sedentary time or light physical activity depending on the predictor. Model 4 was an additional adjustment for either light physical activity or moderate to vigorous physical activity depending on the predictor. Model 5 was the exclusion of lean mass from Model 4 due to its strong relationship with blood pressure.[15] For categorical MVPA variable analyses, all the above-listed covariates were adjusted for in Model 1, whereas in Model 2 lean mass was excluded. A different GLMM was conducted for each sex and sex-specific analyses were not adjusted for sex.

Iso-temporal substitution longitudinal analyses

To simulate the theoretical effect of replacing time spent sedentary with an equal amount of time spent in LPA and MVPA, all activity variables (LPA and MVPA), except for ST, were entered into the GLMM simultaneously, along with a total wear time variable and the above-listed covariates. By including the total wear time variable (the sum of ST, LPA, and MVPA), time is considered isotemporal and hence the regression estimate, for each activity variable in the model reflects the effect of the substitution.[26] The isotemporal substitution differs from the above GLMM single and partition models because the total activity wear time variable is now included in the analysis. For example, cumulative

systolic BP = (β0) cumulative LPA + (β1) cumulative MVPA + (β2) cumulative total activity wear time + (β3) covariates whereas β0 in the formula represents the effect on the outcome indicator caused by replacing ST with LPA per unit of time.[26] Before running these models, the accelerometer-measured continuous variables were rescaled so that a one-unit increase reflected 10 minutes/day for each variable. In the first part of the GLMM analyses, 10-minute bouts of activity were chosen to allow for comparison with previous studies and as suggested as the minimum time-bout accumulation required to achieve current PA recommendations.[14,27] We also analyzed a 30-minute ST substitution with an equal amount of LPA and MVPA to ascertain whether higher doses of PA would theoretically confer more health benefits i.e. lowering BP. We examined two models; Model 1 was a fully adjusted model and Model 2 was Model 1 without adjusting for lean mass.

Mediation path longitudinal analyses

Lastly, mediating path analyses using structural equation models separately examined the mediating role of cumulative fat mass, lean mass, insulin resistance, lipids, and hsCRP on the longitudinal associations of cumulative ST, LPA, or MVPA with each of systolic and diastolic BP. The mediation analysis was conducted in line with the Guideline for Reporting Mediation Analyses of Randomized Trials and Observational Studies (AGReMA).[28] The examined mediation mechanism between ST and BP is partly based on previous studies in which higher ST was associated with worsening adiposity and insulin resistance with the latter, in turn, associated with an increased risk of elevated BP.[14,27,29–31] Analyses were adjusted for age, sex, HDL-c, LDL-c, triglyceride, hsCRP, family history of hypertension and cardiovascular diseases, smoking status, heart rate, glucose, insulin, ST, LPA, MVPA, total fat mass, or lean mass depending on the predictor or mediator. The assumed relationships between exposures, outcomes, and confounders are portrayed in the directed acyclic graph in Supplemental Figure 2. The path models had three equations per regression analysis: the longitudinal associations of cumulative ST, LPA, or MVPA with cumulative total fat mass, lean mass, insulin resistance, lipids, or inflammation (Equation 1); the longitudinal associations of cumulative total fat mass, lean mass,

insulin resistance, lipids or inflammation with systolic or diastolic BP (Equation 2); and the longitudinal associations of cumulative ST, LPA, and MVPA with cumulative systolic or diastolic BP (Equation 3, total effect), and Equation 3'(direct effect) accounted for the mediating role of cumulative total fat mass, lean mass, insulin resistance, lipids or inflammation on the longitudinal associations of cumulative ST, LPA, and MVPA with cumulative systolic or diastolic BP. The proportion of mediating or suppressing roles was estimated as the ratio of the difference between Equation 3 and Equation 3' or the multiplication of Equations 1 and 2 divided by Equation 3 and expressed in percentage. A mediating or indirect role is confirmed when there are statistically significant associations between (a) the predictor and mediator, (b) the predictor and outcome, (c) the mediator and outcome, and when (d) the longitudinal association between the predictor and outcome variable was attenuated upon inclusion of the mediator.[32] However, when the magnitude of the longitudinal association between the predictor and outcome is increased upon inclusion of a third variable, a suppression is confirmed.[32] This means that suppression occurs when the mediational path has an opposite effect, i.e. instead of a decrease in the point estimate of the direct effect between an exposure and an outcome in relation to the total effect, there is rather an increase in the direct effect above the total effect's point estimate.[32] We considered a statistically significant mediation or suppression of <1% as minimal, and ≥1% as partial. Path analyses were conducted with 1,000 bootstrapped samples.[33,34]

Collinearity diagnoses were performed and accepted results with a variance inflation factor <2, considered differences and associations with a 2-sided p-value <0.05 as statistically significant, and concluded based on effect estimates and their confidence intervals (CI). Covariates were identified based on previous studies and are portrayed in the directed acyclic graph (Supplemental Figure 2).[14,19,22,27,31,35–38] We applied Sidak-correction for potential multiple comparisons. Analyses involving 20% of a sample of 10,000 ALSPAC children at 0.8 statistical power, 0.05 alpha, and 2-sided p-value would show a minimum detectable effect size of 0.062 standard deviations if they had relevant exposure for a normally distributed quantitative variable.[39] We also presented a complete case analysis results to inform of bias due to attrition and also to check

for consistency in a rather smaller size (n = 427) that had all predictors, outcomes and covariates at all time points during the 13-year follow-up. Using compositional data analyses,[40] the longitudinal relationships between isometric log-ratio transformed movement behaviours relative to each other and BP was examined and presented in the supplemental appendix. All statistical analyses were performed using SPSS statistics software, Version 27.0 (IBM Corp, Armonk, NY, USA), and mediation analyses structural equation modeling was conducted using IBM AMOS version 27.0.

References:

S1. Harris PA, Taylor R, Minor BL, et al. The REDCap consortium: Building an international community of software platform partners. *J Biomed Inform*. 2019;95:103208. doi:10.1016/j.jbi.2019.103208

S2. Boyd A, Golding J, Macleod J, et al. Cohort profile: The 'Children of the 90s'-The index offspring of the avon longitudinal study of parents and children. *Int J Epidemiol*. 2013;42(1):111-127. doi:10.1093/ije/dys064

S3. Fraser A, Macdonald-wallis C, Tilling K, et al. Cohort profile: The avon longitudinal study of parents and children: ALSPAC mothers cohort. *Int J Epidemiol*. 2013;42(1):97-110. doi:10.1093/ije/dys066

S4. Northstone K, Lewcock M, Groom A, et al. The Avon Longitudinal Study of Parents and Children (ALSPAC): an update on the enrolled sample of index children in 2019. *Wellcome Open Res*. 2019;4:51. doi:10.12688/wellcomeopenres.15132.1

S5. Agbaje AO. Longitudinal Mediating effect of Fatmass and Lipids on Sedentary Time, Light PA, and MVPA with Inflammation in Youth. *J Clin Endocrinol Metab*. 2023;108(12):3250–3259. doi:10.1210/clinem/dgad354

S6. Agbaje AO. Associations of accelerometer-based sedentary time, light physical activity and moderate-to-vigorous physical activity with resting cardiac structure and function in adolescents according to sex, fat mass, lean mass, BMI, and hypertensive status. *Scand J Med Sci Sports*. 2023;33(8):1399-1411. doi:10.1111/sms.14365

S7. Agbaje AO, Perng W, Tuomainen TP. Effects of Accelerometer-based Sedentary Time and Physical Activity on DEXA-measured Fat Mass in 6059 Children. *Nat Commun*. 2023;14:8232. doi:10.1038/s41467-023-43316-w

S8. Agbaje AO. Associations of Sedentary Time and Physical Activity from Childhood with Lipids: A 13-Year Mediation and Temporal Study. *J Clin Endocrinol Metab*. Published online 2023:dgad688. doi:10.1210/clinem/dgad688

S9. Robusto KM, Trost SG. Comparison of three generations of ActiGraph™ activity monitors in children and adolescents. *J Sports Sci*. 2012;30(13):1429-1435. doi:10.1080/02640414.2012.710761

S10. Troiano RP, Berrigan D, Dodd KW, Mâsse LC, Tilert T, Mcdowell M. Physical activity in the United States measured by accelerometer. *Med Sci Sports Exerc*. 2008;40(1):181-188. doi:10.1249/mss.0b013e31815a51b3

S11. Trost SG, Loprinzi PD, Moore R, Pfeiffer KA. Comparison of accelerometer cut points for predicting activity intensity in youth. *Med Sci Sports Exerc*. 2011;43(7):1360-1368. doi:10.1249/MSS.0b013e318206476e

S12. Migueles JH, Cadenas-Sanchez C, Ekelund U, et al. Accelerometer Data Collection and Processing Criteria to Assess Physical Activity and Other Outcomes: A Systematic Review and Practical Considerations. *Sport Med*. 2017;47(9):1821-1845. doi:10.1007/s40279-017-0716-0

S13. Wijndaele K, White T, Andersen LB, et al. Substituting prolonged sedentary time and cardiovascular risk in children and youth: a meta-analysis within the International Children's Accelerometry database (ICAD). *Int J Behav Nutr Phys Act*. 2019;16(1):96. doi:10.1186/s12966-019-0858-6

S14. Bull FC, Al-Ansari SS, Biddle S, et al. World Health Organization 2020 guidelines on physical activity and sedentary behaviour. *Br J Sports Med*. 2020;54(24):1451-1462. doi:10.1136/bjsports-2020-102955

S15. Agbaje AO, Barker AR, Tuomainen TP. Cumulative muscle mass and blood pressure but not fat mass drives arterial stiffness and carotid intima-media thickness progression in the young population and is unrelated to vascular organ damage. *Hypertens Res*. 2023;46:984-999. doi:10.1038/s41440-022-01065-1

S16. Agbaje AO. Elevated Blood Pressure and Worsening Cardiac Damage During Adolescence. *J Pediatr*. 2023;257:113374. doi:10.1016/j.jpeds.2023.02.018

S17. Agbaje AO, Barker AR, Tuomainen TP. Effects of Arterial Stiffness and Carotid Intima- Media Thickness Progression on the Risk of Overweight/Obesity and Elevated Blood Pressure/ Hypertension: a Cross-Lagged Cohort Study. *Hypertension*. 2022;79(1):159-169. doi:10.1161/HYPERTENSIONAHA.121.18449

S18. Agbaje AO, Zachariah JP, Tuomainen TP. Arterial stiffness but not carotid intima-media thickness progression precedes premature structural and functional cardiac damage in youth: A 7-year temporal and mediation longitudinal study. *Atherosclerosis*. 2023;380:117197. doi:10.1016/j.atherosclerosis.2023.117197

S19. Agbaje AO, Barker AR, Mitchell GF, Tuomainen TP. Effect of arterial stiffness and carotid intima-media thickness progression on the risk

of dysglycemia, insulin resistance, and dyslipidaemia: a temporal causal longitudinal study. *Hypertension*. 2022;79(3):667–678. doi:10.1161/HYPERTENSIONAHA.121.18754

S20. Agbaje AO. The Interactive Effects of Sedentary Time, Physical Activity, and Fat Mass on Insulin Resistance in the Young Population. *J Clin Endocrinol Metab*. 2024;(in print). doi:10.1210/clinem/dgae135

S21. Frysz M, Howe LD, Tobias JH, Paternoster L. Using SITAR (Superimposition by translation and rotation) to estimate age at peak height velocity in avon longitudinal study of parents and children [version 2; referees: 2 approved]. *Wellcome Open Res*. 2018;3:90. doi:10.12688/wellcomeopenres.14708.2

S22. Agbaje AO, Barker AR, Tuomainen TP. Cardiorespiratory Fitness, Fat Mass, and Cardiometabolic Health with Endothelial Function, Arterial Elasticity, and Stiffness. *Med Sci Sport Exerc*. 2022;54(1):141-152. doi:10.1249/mss.0000000000002757

S23. Rubin DB. An Overview of Multiple Imputation. *Proc Surv Res methods Sect Am Stat Assoc*. 1988;16:79–84.

S24. Mackinnon A. The use and reporting of multiple imputation in medical research - a review. *J Intern Med*. 2010;268(6):586–593. doi:10.1111/j.1365-2796.2010.02274.x

S25. Schielzeth H, Dingemanse NJ, Nakagawa S, et al. Robustness of linear mixed-effects models to violations of distributional assumptions. *Methods Ecol Evol*. 2020;11:1141– 1152. doi:10.1111/2041-210X.13434

S26. Mekary RA, Willett WC, Hu FB, Ding EL. Isotemporal substitution paradigm for physical activity epidemiology and weight change. *Am J Epidemiol*. 2009;170(4):519–527. doi:10.1093/aje/kwp163

S27. DiPietro L, Al-Ansari SS, Biddle SJH, et al. Advancing the global physical activity agenda: recommendations for future research by the 2020 WHO physical activity and sedentary behavior guidelines development group. *Int J Behav Nutr Phys Act*. 2020;17(1):143. doi:10.1186/s12966-020-01042-2

S28. Lee H, Cashin AG, Lamb SE, et al. A Guideline for Reporting Mediation Analyses of Randomized Trials and Observational Studies: The AGReMA Statement. *JAMA*. 2021;326(11):1045-1056. doi:10.1001/jama.2021.14075

S29. Yaribeygi H, Maleki M, Sathyapalan T, Jamialahmadi T, Sahebkar A. Pathophysiology of Physical Inactivity-Dependent Insulin Resistance: A Theoretical Mechanistic Review Emphasizing Clinical Evidence. *J Diabetes Res*. 2021;2021:7796727. doi:10.1155/2021/7796727

S30. Kerr NR, Booth FW. Contributions of physical inactivity and sedentary behavior to metabolic and endocrine diseases. *Trends Endocrinol Metab*. 2022;33(12):817-827. doi:10.1016/j.tem.2022.09.002

S31. Hill JO, Wyatt HR. Role of physical activity in preventing and treating obesity. *J Appl Physiol*. 2005;99(2):765-770. doi:10.1152/japplphysiol.00137.2005

S32. MacKinnon DP, Krull JL, Lockwood CM. Equivalence of the mediation, confounding and suppression effect. *Prev Sci*. 2000;1(4):173-181. doi:10.1023/a:1026595011371

S33. Preacher KJ, Hayes AF. Asymptotic and resampling strategies for assessing and comparing indirect effects in multiple mediator models. *Behav Res Methods*. 2008;40:879-891. doi:10.3758/BRM.40.3.879

S34. Preacher KJ, Hayes AF. SPSS and SAS procedures for estimating indirect effects in simple mediation models. *Behav Res Methods, Instruments, Comput*. 2004;36:717-731. doi:10.3758/BF03206553

S35. Agbaje AO, Barmi S, Sansum KM, Baynard T, Barker AR, Tuomainen TP. Temporal longitudinal associations of carotid-femoral pulse wave velocity and carotid intima-media thickness with resting heart rate and inflammation in youth. *J Appl Physiol*. 2023;134(3):657-666. https://doi.org/10.1152/japplphysiol.00701.2022

S36. Agbaje AO, Zachariah JP, Bamsa O, Odili AN, Tuomainen TP. Cumulative insulin resistance and hyperglycaemia with arterial stiffness and carotid IMT progression in 1779 adolescents: A 9-Year Longitudinal Cohort Study. *Am J Physiol Endocrinol Metab*. 2023;324(3):E268-E278. doi:10.1152/ajpendo.00008.2023

S37. Agbaje AO. Mediating role of body composition and insulin resistance on the association of arterial stiffness with blood pressure among adolescents: The ALSPAC study. *Front Cardiovasc Med*. 2022;9:939125. doi:10.3389/fcvm.2022.939125

S38. Agbaje AO. Increasing lipids with risk of worsening cardiac damage in 1595 adolescents: A 7-year longitudinal and mediation study. *Atherosclerosis*. Published online 2023:117440. doi:https://doi.org/10.1016/j.atherosclerosis.2023.117440

S39. Golding G, Pembrey P, Jones J. ALSPAC - The Avon Longitudinal Study of Parents and Children I. Study methodology. *Paediatr Perinat Epidemiol*. 2001;15(1):74-87. doi:10.1046/j.1365-3016.2001.00325.x

S40. Egozcue J, Pawlowsky-Glahn V, Mateu-Figueras G, Barcelo-Vidal C. Isometric logratio transformations for compositional data analysis. *Math Geol*. 2003;35(3):279-300.

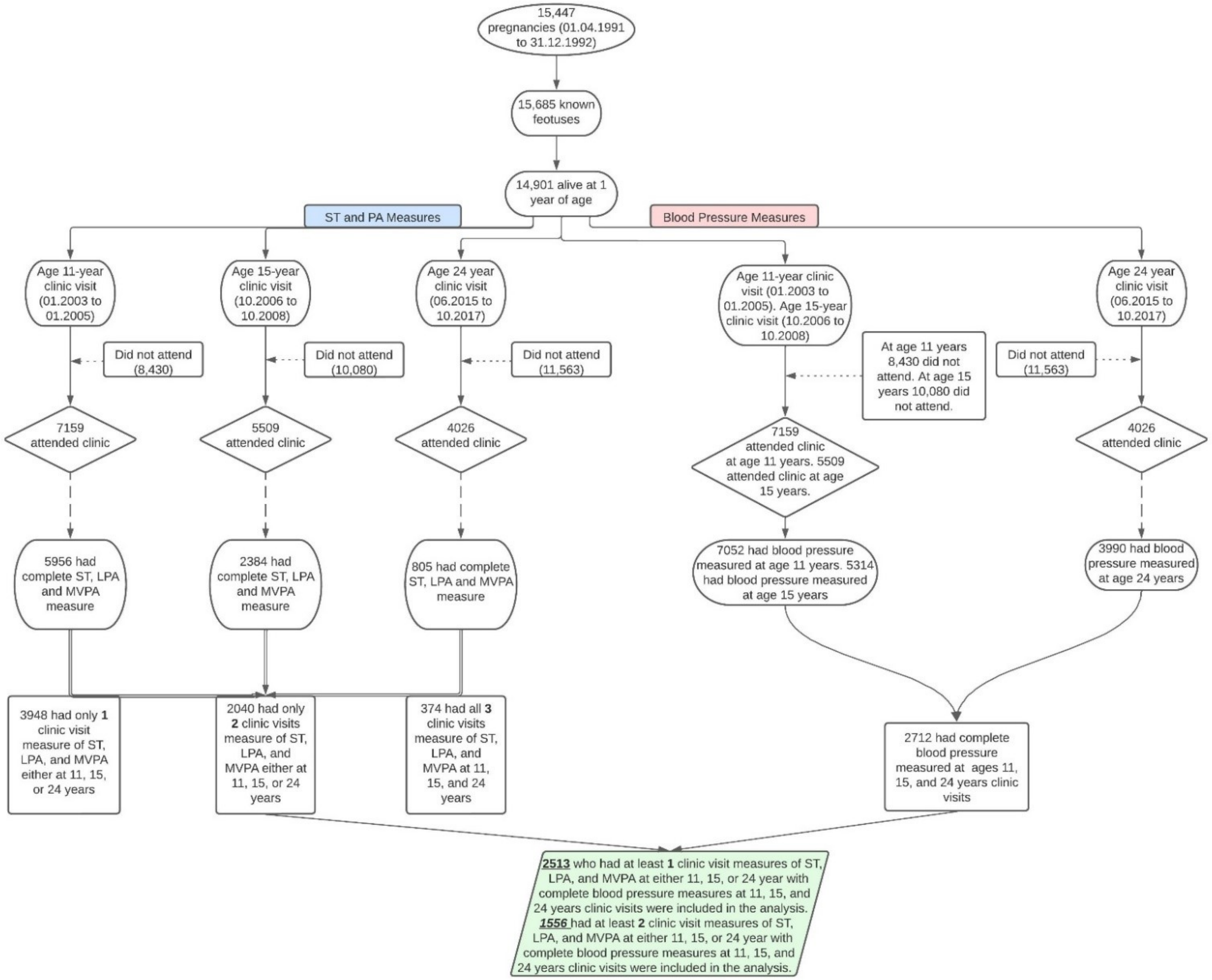

Supplemental Figure 1 Flowchart of cohort participants.

LPA, light physical activity; MVPA, moderate-to-vigorous physical activity; ST, sedentary time. Altogether 5217 participants attended the age 17-year clinic visits between October 2008 and December 2010 of which 4953 participants had valid blood sample measures.

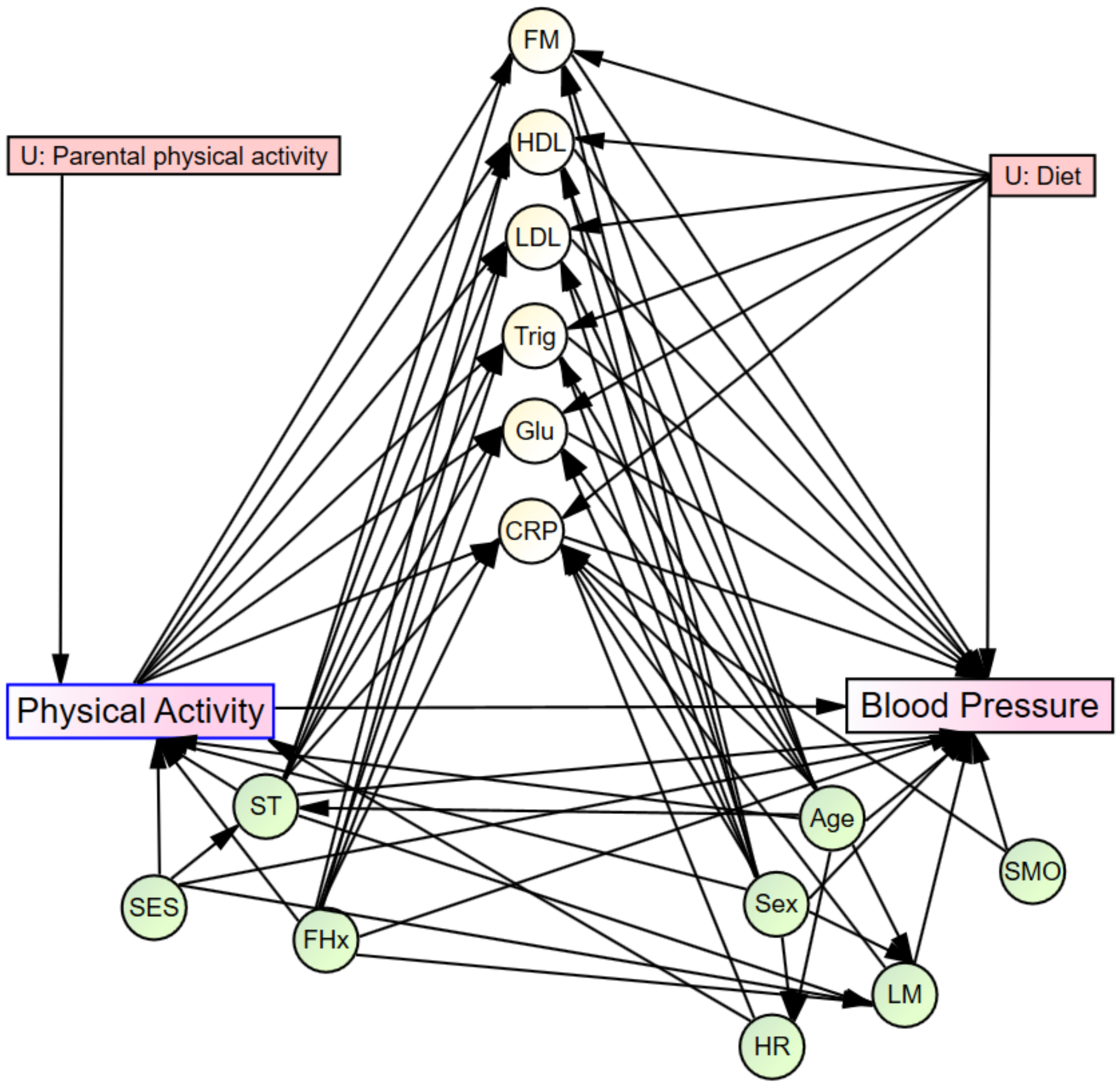

Supplemental Figure 2 Directed Acyclic Graph on the potential causal relationship between physical activity and blood pressure, illustrating mediators (golden colour), confounders (green colour) and unmeasured confounders (red colour). CRP, high sensitivity C-reactive protein; FHx, family history of cardiovascular, cholesterol, diabetes, and hypertensive diseases; FM, fat mass; HDL, high-density lipoprotein cholesterol; HR, heart rate; Glu, glucose or insulin; LDL, low-density lipoprotein cholesterol; LM, lean mass; SMO, smoking; ST, sedentary time; Trig, triglyceride; U, Unmeasured covariates.

Supplemental Table 1 Characteristics of participants excluded from the study based on lack of complete three-time-point blood pressure measurement at age 11-, 15-, and 24-years clinic visit using the age 15-year clinic visit profile.

Variables	**Included participants (n = 2513)**	**Excluded participants (n = 3841)**		
	Mean (SD)	Mean (SD)	*P-value* for difference	Cohen's D
Age (years)	15.39 (0.25)	15.47 (0.33)	**<0.001**	0.25
Anthropometry and body composition				
Body height (m)	1.68 (0.08)	1.70 (0.08)	**<0.001**	0.19
*Weight (kg)	59.70 (12.9)	60.70 (13.7)	**<0.001**	0.08
*Body mass index (kg/m^2)	20.77 (3.78)	20.72 (3.88)	0.657	NA
*Lean mass (kg)	40.49 (12.34)	44.03 (13.90)	**<0.001**	0.27
*Total fat mass (kg)	14.35 (10.77)	12.92 (12.26)	**<0.001**	0.12
Metabolic profile				
HDL (mmol/L)	1.30 (0.29)	1.28 (0.29)	**0.047**	0.07
LDL (mmol/L)	2.09 (0.56)	2.10 (0.56)	0.874	NA
*Triglyceride (mmol/L)	0.75 (0.38)	0.73 (0.40)	0.452	NA
*C-reactive protein (mg/L)	0.37 (0.64)	0.41 (0.78)	**0.002**	0.06
*Insulin (mU/L)	9.05 (5.15)	8.96 (5.33)	0.986	NA
Glucose (mmol/L)	5.20 (0.40)	5.22 (0.39)	0.278	NA
Vascular measure				
Heart rate (beats/mins)	75 (12)	74 (13)	**0.002**	0.09
Systolic blood pressure (mm Hg)	123 (11)	123 (11)	0.523	NA
Diastolic blood pressure (mm Hg)	67 (9)	68 (9)	0.244	NA
Lifestyle factors				
Smoking status (n, %)	332 (13.5)	410 (19.9)	<0.001	0.09†
Family history of HDCV (n, %)	654 (29.8)	519 (30.6)	0.622	NA

The values are means (standard deviations) and *median (range/interquartile range) except for smoking status and family history in percentage. Differences between participants were tested using Student's t-test for normally distributed continuous variables, Mann–Whitney U test for skewed continuous variables, and Chi-square test for dichotomous variables. A 2-sided P-value <0.05 is considered statistically significant and is bolded. Cohen's D effect size was calculated for statistically significant differences in continuous variables: 0.2 = small, 0.5 = moderate, 0.8 = large effect. †Phil and Cramer's V effect size was calculated for statistically significant differences in categorical variables: ≤0.2 = weak, 0.2 - ≤0.6 = moderate, >0.6 = strong effect. HDCV, hypertension/diabetes/high cholesterol/vascular disease; NA, not applicable; Smoking status, participants had smoked cigarettes in the past 30 days.

Supplemental Table 2 Sex-specific longitudinal associations of cumulative sedentary time and physical activity with blood pressure progression from ages 11 through 24 years of 2513 participants who had at least one-time point movement behaviour measure and complete three-time point blood pressure measure

	**Systolic blood pressure**		**Diastolic blood pressure**			**Systolic blood pressure**		**Diastolic blood pressure**	
	***β** (95% CI)*	*p-value*	***β** (95% CI)*	*p-value*		***β** (95% CI)*	*p-value*	***β** (95% CI)*	*p-value*
**Male (n = 976)**						**Female (n = 1537)**			
**Sedentary Time (mins/day)**									
Model 1	0.005 (0.001 – 0.008)	**0.007**	0.006 (0.003 – 0.009)	**<0.001**		0.012 (0.009 – 0.015)	**<0.0001**	0.008 (0.006 – 0.010)	**<0.0001**
Model 2	0.041 (0.036 – 0.046)	**<0.0001**	0.022 (0.018 – 0.025)	**<0.0001**		0.022 (0.019 – 0.025)	**<0.0001**	0.012 (0.010 – 0.014)	**<0.0001**
**Light Physical Activity (mins/day)**									
Model 1	0.000 (-0.004 – -0.004)	0.812	0.000 (-0.004 – 0.003)	0.829		-0.011 (-0.015 – -0.007)	**<0.0001**	-0.002 (-0.005 – 0.001)	0.247
Model 2	0.006 (0.000 – 0.012)	**0.043**	0.002 (-0.001 – 0.006)	0.229		-0.010 (-0.014 – -0.006)	**<0.0001**	-0.001 (-0.004 – 0.002)	0.340
**Moderate to Vigorous Physical Activity (mins/day)**									
Model 1	-0.004 (-0.013 – 0.005)	0.365	0.008 (0.003 – 0.014)	**0.003**		0.000 (-0.009 – 0.009)	0.967	-0.006 (-0.014 – 0.001)	0.104
Model 2	-0.005 (-0.016 – 0.007)	0.428	0.008 (0.001 – 0.015)	**0.018**		0.001 (-0.009 – 0.0011)	0.848	-0.006 (-0.014 – 0.002)	0.135

Model 1 was adjusted for time-varying covariates measured at both baseline and follow-up such as age, low-density lipoprotein cholesterol, triglyceride, high sensitivity C-reactive protein, high-density lipoprotein cholesterol, heart rate, glucose, insulin, smoking status, family history of hypertension/diabetes/high cholesterol/vascular disease, socioeconomic status, fat mass, and lean mass, with additional adjustments for sedentary time (ST), light physical activity (LPA) or moderate to vigorous physical activity (MVPA) depending on the predictor. Model 2 was Model 1 without adjustment for lean mass. Skewed covariates were logarithmically transformed. Unstandardized regression coefficients ***(β)*** were computed from generalized linear mixed-effect model for repeated measures; CI, confidence interval. A 2-sided P-value <0.05 is considered statistically significant. Multiple testing was corrected with Sidak correction. Multiple imputations were used to account for missing variables. A 1-minute change in ST, LPA, and MVPA is associated with a 1-mmHg change in the blood pressure.

Supplemental Table 3 Longitudinal associations of cumulative sedentary time and physical activity with blood pressure progression from ages 11 through 24 years among participants with at least two-time point movement behaviour measure and complete three-time point blood pressure measure.

**N = 1556**	**Systolic blood pressure**		**Diastolic blood pressure**	
	β *(95% CI)*	*p-value*	***β*** *(95% CI)*	*p-value*
Continuous cumulative predictor variables from ages 11 – 24 years				
**Sedentary Time (mins/day)**				
Model 1	0.010 (0.006 – 0.013)	**<0.0001**	0.010 (0.007 – 0.012)	**<0.0001**
Model 2	0.033 (0.028 – 0.037)	**<0.0001**	0.018 (0.015 – 0.021)	**<0.0001**
**Light Physical Activity (mins/day)**				
Model 1	-0.008 (-0.012 – -0.004)	**<0.0001**	-0.003 (-0.007 – 0.000)	0.050
Model 2	-0.006 (-0.011 – 0.000)	**0.042**	-0.003 (-0.006 – 0.001)	0.173
**Moderate to Vigorous Physical Activity (mins/day)**				
Model 1	-0.004 (-0.014 – 0.007)	0.513	0.004 (-0.003 – 0.011)	0.291
Model 2	-0.026 (-0.040 – -0.013)	**<0.0001**	-0.004 (-0.012 – 0.004)	0.328
Categorical cumulative predictor variable from ages 11 – 24 years				
**Moderate to Vigorous Physical Activity**				
Model 1	***<40mins/day of MVPA as reference***			
40 – <60mins/day	-0.111 (-0.622 – 0.400)	0.671	-0.168 (-0.588 – 0.734)	0.390
≥60mins/day	-0.076 (-0.710 – 0.559)	0.815	0.224 (-0.286 – 0.705)	0.432
Model 2	***<40mins/day of MVPA as reference***			
40 – <60mins/day	-0.647 (-1.293 – -0.001)	0.050	-0.358 (-0.797 – 0.082)	0.110
≥60mins/day	-1.615 (-2.435 – -0.796)	**<0.0001**	-0.323 (-0.861 – 0.215)	0.239

Model 1 was adjusted for sex and time-varying covariates measured at both baseline and follow-up such as age, low-density lipoprotein cholesterol, triglyceride, high sensitivity C-reactive protein, high-density lipoprotein cholesterol, heart rate, glucose, insulin, smoking status, family history of hypertension/diabetes/high cholesterol/vascular disease, socioeconomic status, fat mass, and lean mass, with additional adjustments for sedentary time (ST), light physical activity (LPA) or moderate to vigorous physical activity (MVPA) depending on the predictor. Model 2 was Model 1 without adjustment for lean mass. Skewed covariates were logarithmically transformed. Unstandardized regression coefficients ***(β)*** were computed from generalized linear mixed-effect model for repeated measures; CI, confidence interval. A 2-sided P-value <0.05 is considered statistically significant. Multiple testing was corrected with Sidak correction. Multiple imputations were used to account for missing variables. A 1-minute change in ST, LPA, and MVPA is associated with a 1-mmHg change in the blood pressure.

Supplemental Table 4 Complete case analysis of longitudinal associations of cumulative sedentary time and physical activity with blood pressure progression from ages 11 through 24 years among participants with at least one-time point movement behaviour measure and complete three-time point blood pressure measure.

**N= 427**	**Systolic blood pressure**		**Diastolic blood pressure**	
	β *(95% CI)*	*p-value*	***β*** *(95% CI)*	*p-value*
**Sedentary Time (mins/day)**				
Model 1	0.049 (0.045 – 0.052)	**<0.0001**	0.032 (0.029 – 0.034)	**<0.0001**
Model 2	0.017 (0.010 – 0.025)	**<0.001**	0.012 (0.006 – 0.017)	**<0.001**
**Light Physical Activity (mins/day)**				
Model 1	-0.043 (-0.047 – -0.040)	**<0.0001**	-0.030 (-0.032 – -0.028)	**<0.0001**
Model 2	0.001 (-0.009 – 0.011)	0.884	0.000 (-0.007 – 0.008)	0.898
**Moderate to Vigorous Physical Activity (mins/day)**				
Model 1	-0.058 (-0.074 – -0.042)	**<0.001**	-0.035 (-0.047 – -0.024)	**<0.001**
Model 2	-0.015 (-0.030 – 0.001)	0.063	-0.002 (-0.007 – 0.008)	0.704

Model 1 was unadjusted. Model 2 was adjusted for sex and time-varying covariates measured at both baseline and follow-up such as age, low-density lipoprotein cholesterol, triglyceride, high sensitivity C-reactive protein, high-density lipoprotein cholesterol, heart rate, glucose, insulin, smoking status, family history of hypertension/diabetes/high cholesterol/vascular disease, socioeconomic status, fat mass, and lean mass, with additional adjustments for sedentary time (ST), light physical activity (LPA) or moderate to vigorous physical activity (MVPA) depending on the predictor. Skewed covariates were logarithmically transformed. Unstandardized regression coefficients ***(β)*** were computed from generalized linear mixed-effect model for repeated measures; CI, confidence interval. A 2-sided P-value <0.05 is considered statistically significant. Multiple testing was corrected with Sidak correction. Multiple imputations were used to account for missing variables. A 1-minute change in ST, LPA, and MVPA is associated with a 1-mmHg change in the blood pressure.

Supplemental Table 5 Compositional data analysis of the longitudinal associations of cumulative sedentary time and physical activity with blood pressure progression from ages 11 through 24 years among participants with at least one-time point movement behaviour and blood pressure measure.

**N = 2513**	**Systolic blood pressure**		**Diastolic blood pressure**	
	β *(95% CI)*	*p-value*	***β*** *(95% CI)*	*p-value*
Continuous cumulative predictor variables from ages 11 – 24 years				
**Sedentary Time relative to LPA and MVPA**				
Model 1	0.002 (-0.003 – 0.006)	0.457	-0.001 (-0.005 – 0.002)	0.402
Model 2	0.008 (0.002 – 0.013)	**0.004**	0.001 (-0.003 – 0.005)	0.595
**LPA relative to Sedentary Time and MVPA**				
Model 1	-0.514 (-0.712 – -0.317)	**<0.001**	-0.497 (-0.655 – -0.339)	**<0.001**
Model 2	-1.251 (-1.506 – -0.997)	**<0.0001**	-0.784 (-0.951 – -0.617)	**<0.0001**
**MVPA relative to Sedentary Time and LPA**				
Model 1	0.000 (-0.004 – -0.004)	0.945	0.003 (-0.001 – 0.006)	0.101
Model 2	-0.004 (-0.010 – 0.001)	0.131	0.001 (-0.002– 0.005)	0.488
**LPA relative to Sedentary Time**				
Model 1	-0.005 (-0.013 – 0.002)	0.155	0.000 (-0.006 – 0.006)	0.995
Model 2	-0.020 (-0.029 – -0.011)	**<0.001**	-0.006 (-0.012 – 0.001)	0.077
**MVPA relative to Sedentary Time**				
Model 1	-0.001(-0.005 – 0.003)	0.686	0.002 (-0.001 – 0.005)	0.213
Model 2	-0.005 (-0.010 – -0.001)	**0.029**	0.000 (-0.003 – 0.003)	0.913

Model 1 was adjusted for sex and time-varying covariates measured at both baseline and follow-up such as age, low-density lipoprotein cholesterol, triglyceride, high sensitivity C-reactive protein, high-density lipoprotein cholesterol, heart rate, glucose, insulin, smoking status, family history of hypertension/diabetes/high cholesterol/vascular disease, socioeconomic status, fat mass, and lean mass, with additional adjustments for sedentary time, light physical activity (LPA) or moderate to vigorous physical activity (MVPA) depending on the predictor. Model 2 was Model 1 without adjustment for lean mass. Skewed covariates were logarithmically transformed. Unstandardized regression coefficients ***(β)*** were computed from generalized linear mixed-effect model for repeated measures; CI, confidence interval. A 2-sided P-value <0.05 is considered statistically significant. Multiple testing was corrected with Sidak correction. Multiple imputations were used to account for missing variables. A 1-minute change in ST, LPA, and MVPA is associated with a 1-mmHg change in the blood pressure.
